# Fanca deficiency is associated with alterations in osteoclastogenesis that are rescued by TNFα

**DOI:** 10.1186/s13578-023-01067-7

**Published:** 2023-06-24

**Authors:** Alessia Oppezzo, Lovely Monney, Henri Kilian, Lofti Slimani, Frédérique Maczkowiak-Chartois, Filippo Rosselli

**Affiliations:** 1 CNRS UMR9019, Équipe labellisée La Ligue contre le Cancer, Gustave Roussy Cancer Campus, 114 rue Edouard Vaillant, 94805 Villejuif, France; 2 Gustave Roussy Cancer Center, Villejuif, France; 3 Université Paris Saclay, Orsay, France; 4 URP2496 Pathologies, Imagerie et Biothérapies Orofaciales et Plateforme Imagerie du Vivant (PIV), FHU-DDS-net, Dental School, Université de Paris, Montrouge, France; 5 Present Address: IFOM ETS, The AIRC Institute of Molecular Oncology, Milan, Italy

**Keywords:** Fanconi anemia, Osteoclast, Osteoblast, Cell signaling, TNFα, p53

## Abstract

**Background:**

Hematopoietic stem cells (HSCs) reside in the bone marrow (BM) niche, which includes bone-forming and bone-resorbing cells, i.e., osteoblasts (OBs) and osteoclasts (OCs). OBs originate from mesenchymal progenitors, while OCs are derived from HSCs. Self-renewal, proliferation and differentiation of HSCs are under the control of regulatory signals generated by OBs and OCs within the BM niche. Consequently, OBs and OCs control both bone physiology and hematopoiesis. Since the human developmental and bone marrow failure genetic syndrome fanconi anemia (FA) presents with skeletal abnormalities, osteoporosis and HSC impairment, we wanted to test the hypothesis that the main pathological abnormalities of FA could be related to a defect in OC physiology and/or in bone homeostasis.

**Results:**

We revealed here that the intrinsic differentiation of OCs from a *Fanca*^*−/−*^ mouse is impaired in vitro due to overactivation of the p53–p21 axis and defects in NF-kB signaling. The OC differentiation abnormalities observed in vitro were rescued by treating *Fanca*^*−/−*^ cells with the p53 inhibitor pifithrin-α, by treatment with the proinflammatory cytokine TNFα or by coculturing them with Fanca-proficient or Fanca-deficient osteoblastic cells.

**Conclusions:**

Overall, our results highlight an unappreciated role of Fanca in OC differentiation that is potentially circumvented in vivo by the presence of OBs and TNFα in the BM niche.

**Supplementary Information:**

The online version contains supplementary material available at 10.1186/s13578-023-01067-7.

## Background

Bone marrow failure (BMF), myelodysplasia (MDS) and acute myeloid leukemia (AML) are three pathological conditions with underlying alterations in hematopoietic stem and progenitor cells (HSPCs) due to the cytotoxic consequences of exogenous stress (DNA damage) as well as to acquired or inherited mutations in genes encoding proteins involved in several classes of biological functions, including epigenetics [1, 2], RNA transcription and/or splicing [3–5], metabolism [6, 7], signaling [8, 9], DNA repair [10–12], ribosome biogenesis [13, 14] and/or telomere maintenance [15, 16].

After birth, HSPCs are localized in a specific microenvironment in the bone marrow (BM), the hematopoietic niche. Niche homeostasis is determined by tightly regulated crosstalk between HSPCs and niche components that regulates the quiescence, proliferation, and differentiation of the former and influences the dynamics of the latter [17, 18]. Indeed, although BMF, MDS and AML have long been considered HSPC-autonomous disorders whose development is driven by cell-intrinsic events, several observations definitively support a key role of the HSPC microenvironment in the initiation, selection, and progression of these three conditions [19–21]. Bone-forming osteoblasts (OBs) originating from mesenchymal stem cells (MSCs) and bone-resorbing osteoclasts (OCs) originating from HSPCs are key components of the niche and are known to have a major influence on both healthy and pathological HSPC behavior [22–25]. Accordingly, analyses of experimental mouse models or human pathologies have shown that specific gene mutations [26, 27] and functional deficiencies in OBs and/or OCs [28–30] are, per se, sufficient to generate BMF, MDS and AML.

Fanconi anemia (FA) is a DNA repair deficiency syndrome linked to BMF and cancer predisposition (especially MDS and AML) [31]. To date, more than 20 genes are known to be mutated in FA, with *FANCA* accounting for more than 60% of all FA cases [32]. FA-related proteins participate in a pathway involved in DNA interstrand crosslink (ICL) repair and in the rescue of stalled replication forks, ensuring maintenance of genetic and chromosomal integrity [33, 34]. However, in addition to their key roles in DNA repair and replication rescue, at least some of the FA-related proteins have other functions, the alteration of which contributes in an as yet poorly determined way to the symptoms of this syndrome. BMF, MDS and AML in FA are associated with DNA damage accumulation and genetic instability but also with alterations in senescence [35, 36]; nucleolar homeostasis and ribosome biogenesis [37–39] as well as in the activity of several stress signaling pathways, such as p38MAPK, PI3K/AKT and NF-kB [40–42]; increased expression or sensitivity to several proinflammatory cytokines, such as TNFα and TGFβ [41, 43, 44]; and the unrestrained expression or activity of p53 [45, 46], Myc [47] and/or microphthalmia transcription factor (MiTF) [48].

In addition to hematopoietic defects, FA patients have a high incidence of mesenchymal tissue-derived congenital malformations and osteoporosis [49–52], suggesting a role of FA-related proteins in osteogenesis and bone maintenance. Despite these clinical observations suggesting multiple mesenchymal defects, relatively little attention has been directed to the association between abnormal HSC functions and the BM microenvironment in FA [49]. Therefore, we wanted to determine whether the process of OC differentiation from precursors (or osteoclastogenesis) was altered in FA.

## Results

### *Fanca* deficiency is associated with altered osteoclastogenesis

**Fig. 1:**
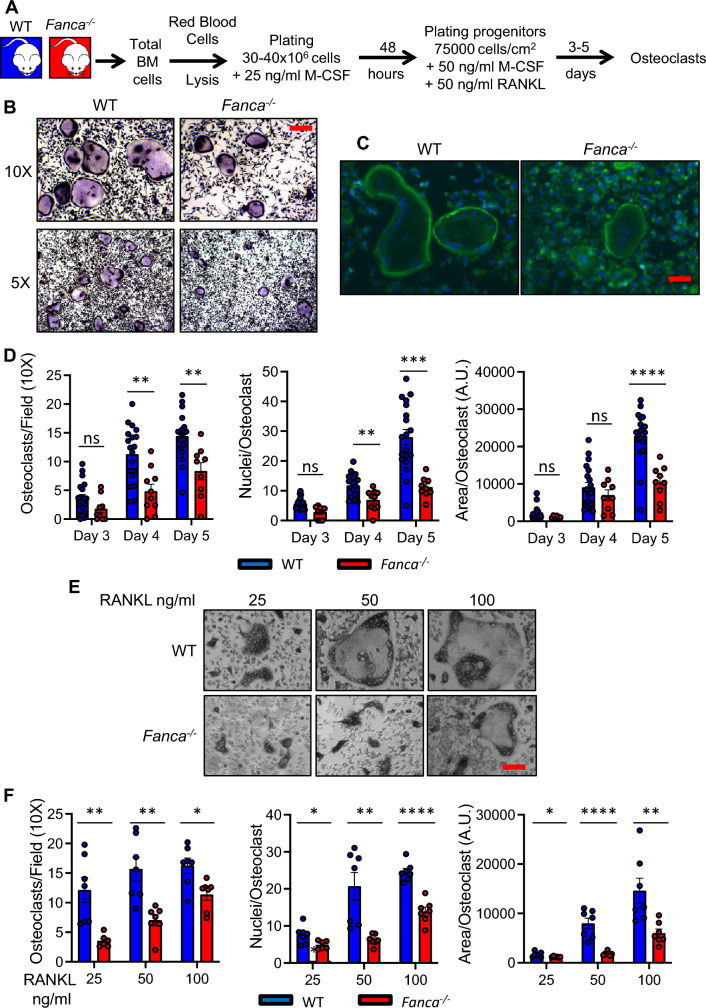
Fanca deficiency is associated with altered osteoclastogenesis. **A** Representation of the strategy used to differentiate OCs from isolated BM cell populations. **B** TRAP staining of OCs from WT and *Fanca*^*−/−*^ mice at day 5 of differentiation. **C** Representative images of F-actin IF in OCs from WT and *Fanca*^*−/−*^ mice at day 5 of differentiation. **D** Number of OCs per field (left), number of nuclei for OC (middle) and surface of the OCs (right) at days 3, 4 and 5 of differentiation. **E** Representative images after TRAP staining of OCs from WT and *Fanca*^*−/−*^ mice at day 4 of differentiation after treatment with the indicated doses of RANKL. **F** Number of OCs per field (left), number of nuclei for OC (middle) and surface of the OCs (right) at day 4 of differentiation after treatment with the indicated doses of RANKL. Data are shown as the mean ± SEM. Each small circle represents an individual mouse. Statistical analysis was performed by t tests: *p < 0.05, **p < 0.01, ***p < 0.001, ****p < 0.0001

To explore the role of Fanca in osteoclastogenesis, we isolated BM cells from 3- to 4-month-old *WT* and *Fanca*^*−/−*^ mice and treated them with macrophage colony stimulating factor (M-CSF) (25 ng/ml) for 48 h to induce OC precursor proliferation. The resulting cells were harvested, counted, resuspended in complete medium supplemented with M-CSF (50 ng/ml) and RANKL (50 ng/ml) and seeded at 75,000 cells/cm^2^ to terminally differentiate into multinucleated OCs (i.e., cells containing at least 3 nuclei) that appeared by day 3 (Fig. 1A). OC formation was monitored by tartrate-resistant acid phosphatase (TRAP) staining (Fig. 1B) or by immunofluorescence (IF) targeting the actin-F cytoskeleton (Fig. 1C). Compared with their WT counterparts, *Fanca*^*−/−*^ cells generated fewer syncytia, each containing fewer nuclei and occupying a smaller area (Fig. 1D, left to right). Comparable results were observed in 1-year-old mice (Additional file 1: Fig. S1A). Next, we demonstrated in WT cells that Fanca expression is maintained without major changes at both the mRNA and protein levels during the differentiation process from precursors to OCs (Additional file 1: Fig. S1B and C). In the knockout (KO) mice, Fanca was undetectable by qPCR and Western blot analysis, confirming gene inactivation and the absence of the protein.

Finally, we analyzed the behavior of the precursors treated with increasing doses of RANKL (25, 50 or 100 ng/ml). At higher doses of RANKL, the cells that merged in a syncytium (Fig. 1F, middle) and the surface of the latter (Fig. 1F, right) increased in both WT and *Fanca*^*−/−*^ cells, whereas the number of OCs per field increased significantly only in *Fanca*^*−/−*^ cells (Fig. 1F, left). Notably, even if osteoclastogenesis in *Fanca*^*−/−*^-derived cells remained significantly lower than that in their WT counterparts when compared at the same dose of RANKL, it appeared that OC differentiation at 100 ng/ml cytokine in *Fanca*^*−/−*^ cells was comparable to that observed at 25 ng/ml in WT cells.

Thus, our in vitro observations robustly demonstrated that Fanca deficiency affects OC differentiation, impacting their fusion and maturation processes.

### *Fanca*^*−/−*^-associated osteoclastogenic abnormalities rely on reduced Nfact1 expression

**Fig. 2:**
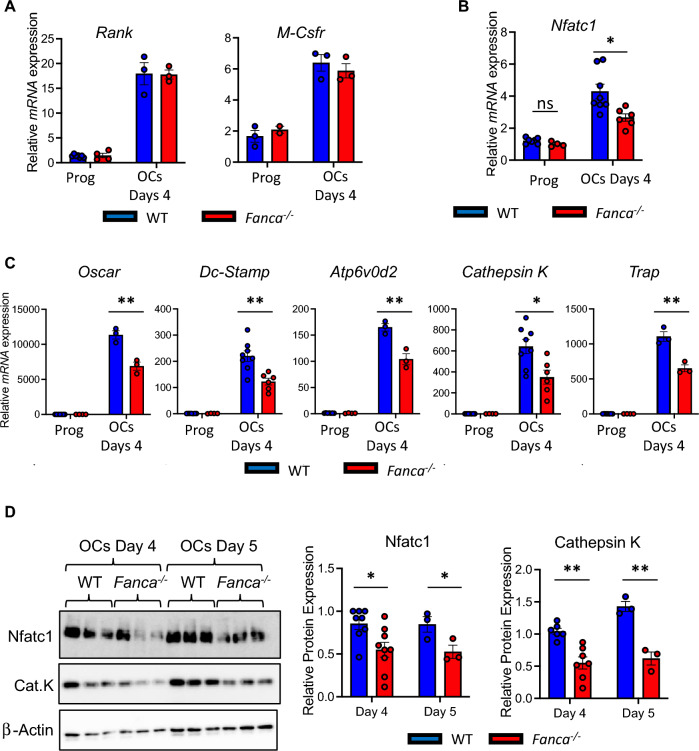
Fanca deficiency leads to impaired osteoclastogenesis at the molecular level. **A** Relative *Rank* and *M-Csfr* expression evaluated by qRT-PCR in progenitors and OCs from WT and *Fanca*^*−/−*^ mice at day 4 of differentiation. **B** Relative *Nfatc1* expression evaluated by qRT-PCR in progenitors and OCs from WT and *Fanca*^*−/−*^ mice at day 4 of differentiation. **C** Relative *Oscar, Dc-Stamp, Atp6v0d2, cathepsin K* and *TRAP* expression evaluated by qRT-PCR in progenitors and OCs from WT and *Fanca*^*−/−*^ mice at day 4 of differentiation. **D** Representative Western blot showing Nfatc1 and Cathepsin K in OCs from WT and *Fanca*^*−/−*^ mice at days 4 and 5 of differentiation. Relative protein expression of Nfatc1 and Cathepsin K in WT and *Fanca*^*−/−*^ mice at days 4 and 5 of differentiation. β-Actin was used as a loading control. Data are shown as the mean ± SEM. Each small circle represents an individual mouse. Statistical analysis was performed by t tests: *p < 0.05, **p < 0.01, ***p < 0.001, ****p < 0.0001

To identify altered partners and/or pathways involved in the *Fanca*^*−/−*^-associated osteoclastogenesis defect, we analyzed the expression of known key elements involved in OC differentiation by qPCR and/or Western blotting. We first demonstrated that the receptors of M-CSF and RANKL, M-Csfr and Rank, respectively, were similarly expressed at the mRNA level in both WT and *Fanca*^*−/−*^ cells (Fig. 2A). Subsequently, we determined the induction of Nfatc1, the key osteogenic transcription factor that activates downstream RANKL exposure and drives OC differentiation [53]. Compared to WT cells, *Fanca*^*−/−*^ OCs exhibited significantly lower Nfatc1 expression at both the mRNA and protein levels (Fig. 2B and D). In accordance with its reduced expression, the mRNA expression of the key Nfatc1 targets [53] Oscar (a marker of OC differentiation), Dc-Stamp and Atp6v0d2 (membrane proteins involved in OC precursor fusion) and Cathepsin K and Trap (involved in bone resorption) was also significantly decreased in *Fanca*^*−/−*^ OCs (Fig. 2C). The decrease in Cathepsin K was also validated at the protein level (Fig. 2D). The same key osteoclastogenic effectors also showed downregulated expression in 1-year-old *Fanca*^*−/−*^ mice (Additional file 1: Fig. S2A and B).

The expression of Mitf, another key osteoclastogenic transcription factor connected to the FANC pathway [54, 55] that we previously described as dysregulated in FA cells [48], was similar between the WT and *Fanca*^*−/−*^ OCs (Additional file 1: Fig. S2C), suggesting that its deregulation in FA is cell type dependent.

Altogether, our analysis indicates that the observed defect in osteoclastogenesis in the absence of Fanca relies on early events linked to the expression/activity of Nfatc1.

### p53 participates in the impaired osteoclastogenesis induced by *Fanca* KO

**Fig. 3:**
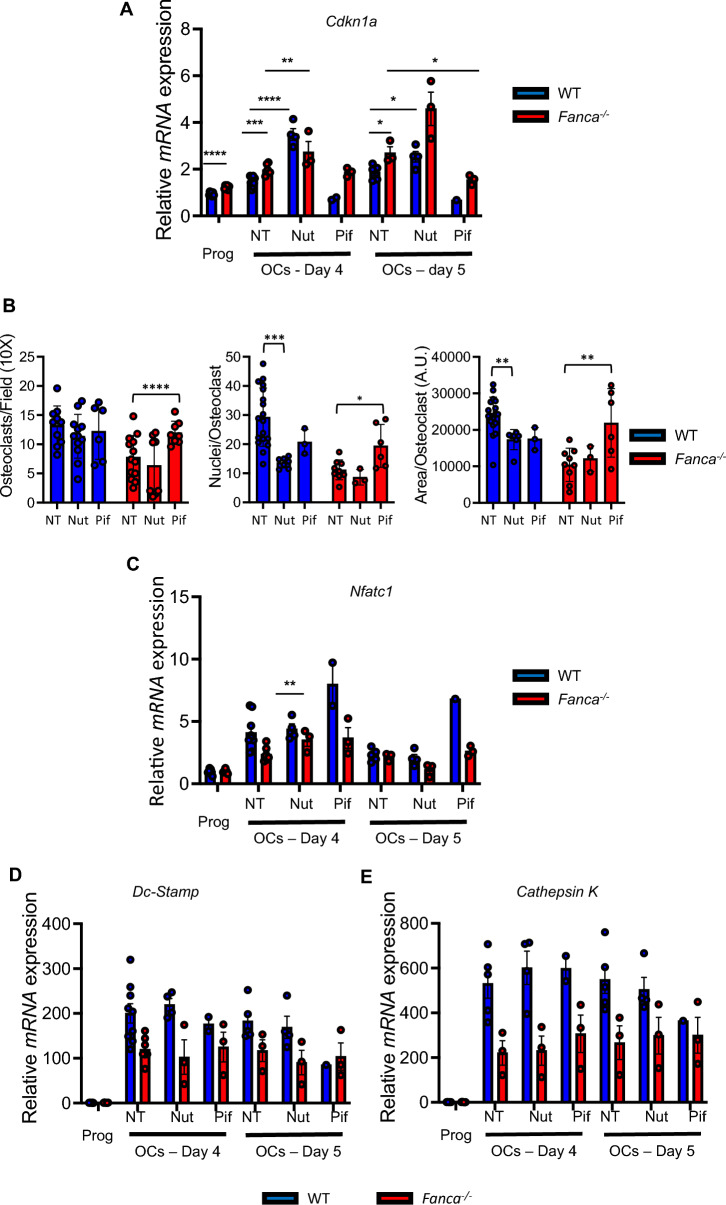
p53–p21 participates in impaired osteoclastogenesis induced by *Fanca* KO. **A** Relative *Cdkn1a* expression evaluated by qRT-PCR in progenitors, OCs and OCs from WT and *Fanca*^*−/−*^ mice treated with Nutlin-3 or pifithrin-α (Nut and Pif, 1 μM each) at days 4 and 5 of differentiation. **B** Number of OCs for field (left), number of nuclei for OC (middle) and surface of the OCs (right) from the WT and *Fanca*^*−/−*^ mice at day 5 of differentiation after Nutlin-3 (Nut) or Pifithrin-α (Pif) treatment (1 μM each). **C**–**E** Relative *Nfatc1, DC-Stamp* and *cathepsin K* expression evaluated by qRT-PCR in progenitors, OCs and OCs from *Fanca*^*−/−*^ mice treated with Nutlin-3 or pifithrin-α (Nut and Pif, 1 μM each) at days 4 and 5 of differentiation. Data are shown as the mean ± SEM. Each small circle represents an individual mouse. Statistical analysis was performed by t tests: *p < 0.05, **p < 0.01, ***p < 0.001, ****p < 0.0001

Previous observations reported an unscheduled constitutive activation of the p53 signaling pathway in cells from FA patients and mice [35, 45, 46] that impacts hematopoietic stem and precursor cell behavior [45]. Since p53 “constitutive” activation in response to its well-known activator Nutlin-3 impairs osteoclastogenesis [56] and activated p53 can potentially lead to downregulated Nfatc1 expression [57], we wanted to monitor OC differentiation from *Fanca*^*−/−*^ cells in the presence of either Nulin-3 or the p53 inhibitor pifithrin-α. First, to validate the impact of each drug on the activity of p53, we quantified the mRNA expression of the key p53 target *Cdkn1a* encoding the cyclin-dependent kinase inhibitor p21 by qPCR. In accordance with the known increased basal level of p53 activity downstream of the loss-of-function of Fanca, *Cdk1a* levels were more elevated in untreated *Fanca*^*−/−*^ than in WT progenitors (Fig. 3A). Next, having validated that in both WT and *Fanca*^*−/−*^ cells, Nutlin-3 exposure increased *Cdkn1a* expression, whereas treatment with pifithrin-α decreased it, we determined OC differentiation in cells exposed to one or the other drug. In WT cells, even if the number of formed OCs remained the same, both p53 activation and inhibition restrained the number of cells that merged in a syncytium and, consequently, their surface (Fig. 3B), suggesting that, at least in vitro, well-regulated p53 activity is necessary for proper osteoclastogenesis. Supporting an inhibitory role of p53 activity in the delayed process of osteoclastogenesis in *Fanca*^*−/−*^ cells, its inhibition by pifithrin-α rescued the number of formed OCs, the number of cells that merged in a syncytium and their surface (Fig. 3B) at a level that is statistically indistinguishable from that of the WT cells under standard conditions. Exposure of *Fanca*^*−/−*^ cells to Nutlin-3 did not modify their behavior. However, despite the improvement in osteoclastogenesis, the expression of the mRNAs encoding Nfatc1, Dc-Stamp and Cathepsin-K was not modified in pifithrin-α-treated *Fanca*^*−/−*^ cells (Fig. 3C).

Thus, although our data support a key role for the unscheduled increase in p53 signaling in the impaired osteoclastogenesis observed in *Fanca*^*−/−*^ cells, it appears that such an effect is likely due to the known effects of p53 on several aspects of cellular behavior, such as the cell cycle, apoptosis and/or senescence, rather than a direct impact on the control of specific pathways involved in OC differentiation.

### TNFα treatment rescues osteoclastogenesis in *Fanca*^*−/−*^ cells by increasing NF-kB activity

**Fig. 4:**
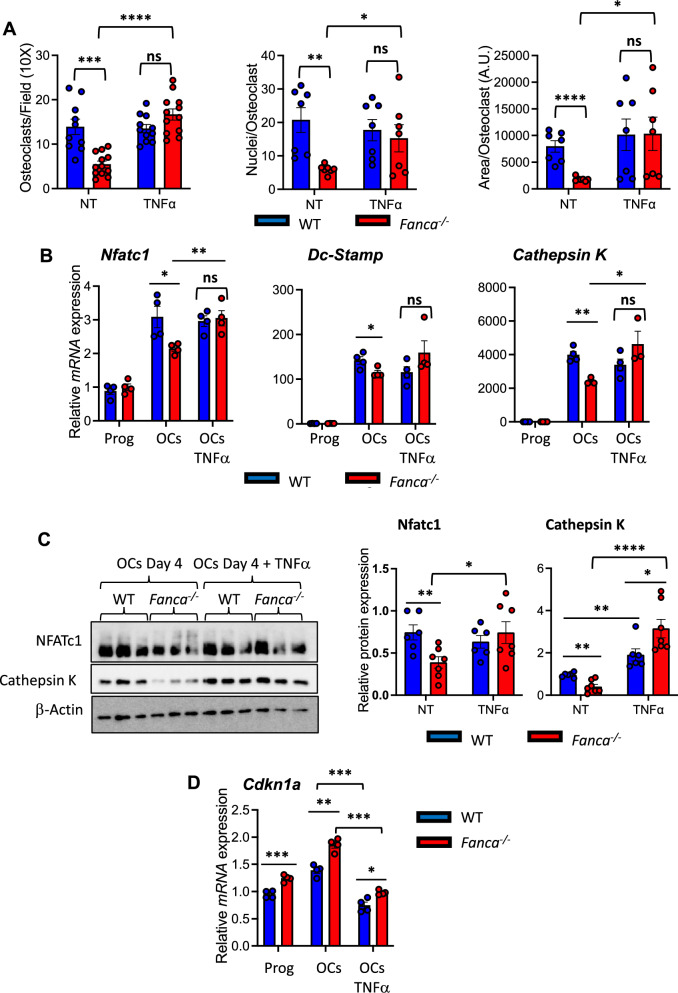
TNFα treatment rescues impaired osteoclastogenesis induced by *Fanca* KO. **A** Number of OCs for field (left), number of nuclei for OC (middle) and surface of the OCs (right) from the WT and *Fanca*^*−/−*^ mice at day 4 of differentiation after TNFα treatment (10 ng/ml). **B** Relative *Nfatc1, Dc-Stamp* and *Cathepsin K* expression evaluated by qRT-PCR in progenitors, OCs and OCs treated with TNFα at day 4 of differentiation from the WT and *Fanca*^*−/−*^ mice. **C** Left: Western blot showing Nfatc1 and Cathepsin K in OCs from WT and *Fanca*^*−/−*^ mice at day 4 of differentiation that were left untreated or treated with TNFα. β-Actin was used as a loading control. Right: Relative protein expression of Nfatc1 and Cathepsin K in OCs from WT and *Fanca*^*−/−*^ mice at day 4 of differentiation that were left untreated or treated with TNFα. **D** Relative *Cdkn1a* expression evaluated by qRT-PCR in progenitors, OCs and OCs treated with TNFα at day 4 of differentiation from WT and *Fanca*^*−/−*^ mice. Data are shown as the mean ± SEM. Each small circle represents an individual mouse. Statistical analysis was performed by t tests: *p < 0.05, **p < 0.01, ***p < 0.001, ****p < 0.0001

TGFβ and TNFα are two extracellular molecules known to regulate osteoclastogenesis [58, 59], and their signaling pathways have been identified as constitutively active, even in the absence of detected cytokine expression, FA [41, 44]. We did not detect either of the two proteins in the culture medium of differentiated OCs from our *Fanca*^*−/−*^ mice as already described in vivo or in cell culture from FANC-KO mouse models (data not shown). Whereas TGFβ exposure had limited consequences on OC differentiation in both WT and *Fanca*^*−/−*^ cells and no effect on the number of scored OCs per field (Additional file 1: Fig. S3), TNFα exposure fully rescued osteoclastogenesis in *Fanca*^*−/−*^ cells without modifying the dynamics of the differentiation process of the WT cells (Fig. 4A). Indeed, TNFα exposure normalized not only the number of OCs per field, the number of nuclei for OC and the area of each syncytium (Fig. 4A) but also the expression of the OC differentiation-associated mRNAs *Nfatc1*, *Dc-Stamp* and *Cathepsin K* (Fig. 4B). Normalization was also validated at the protein level for Nfatc1 and cathepsin K (Fig. 4C). Moreover, we also observed that the expression of *Cdkn1a* was significantly downregulated in *Fanca*^*−/−*^ cells differentiated in the presence of TNFα (Fig. 4D), as previously observed following pifithrin-α treatment, suggesting that TNFα exposure opposes the consequences of unscheduled p53–p21 axis activity that negatively influences the behavior of *Fanca*^*−/−*^ OCs.

**Fig. 5:**
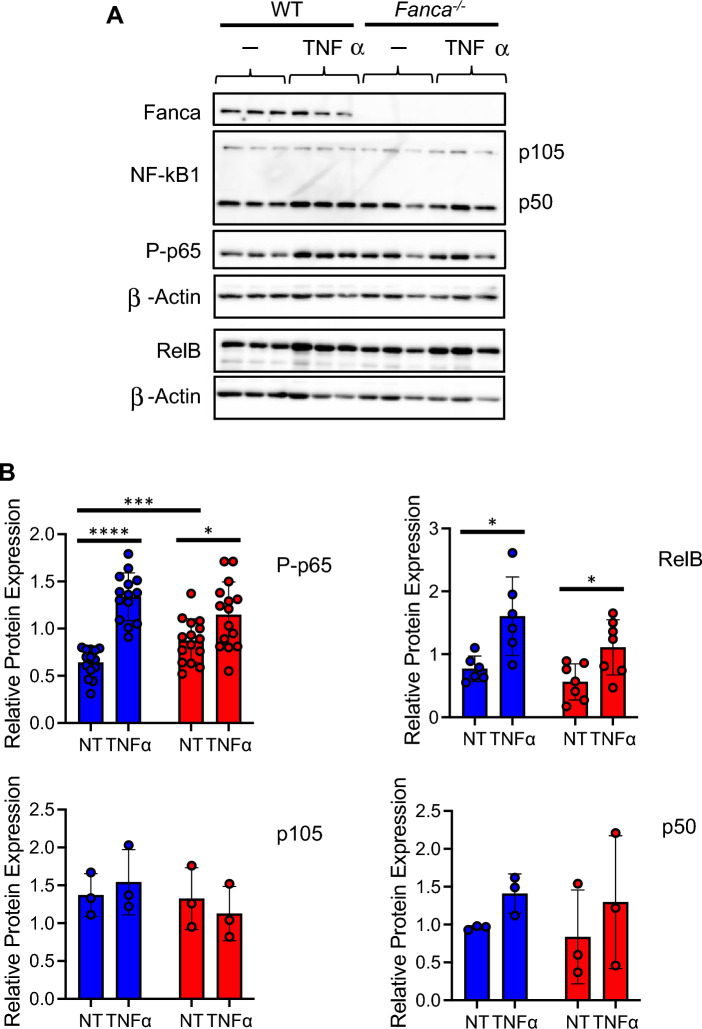
NF-kB status in OCs from WT and *Fanca*^*−/−*^ mice with or without TNFα. **A** Representative Western blot showing NF-kB canonical and noncanonical signaling pathway protein levels 4 days postdifferentiation in OCs from WT and *Fanca*^*−/−*^ mice with or without TNFα. β-Actin was used as a loading control. **B** Quantification of data concerning P-p65, p105, p50 and RelB 4 days postdifferentiation in OCs from WT and *Fanca*^*−/−*^ mice left untreated (NT) or treated with TNFα. Blue bars are for WT and red bars for *Fanca*^-/-^ OCs. Data are shown as the mean ± SEM. Each small circle represents an individual mouse. Statistical analysis was performed by t tests: *p < 0.05, **p < 0.01, ***p < 0.001, ****p < 0.0001

TNFα exposure stimulates NF-kB signaling, which participates in osteoclastogenesis fostering Nfatc1 activity [60]. To indirectly evaluate NF-kB activity in *Fanca*^*−/−*^ OCs in response to TNFα we determined the level of phosphorylation of RelA/p65 and the expression of RelB, both of which are able to interact with p50, cleaved from the p105 precursor, forming dimers that constitute the transcriptionally active forms of the canonical NF-kB pathway that are specifically induced by the cytokine. At 4 days of differentiation, the levels of P-p65 and RelB were significantly more elevated in *Fanca*^*−/−*^ OCs treated with TNFα than in their absence, supporting the hypothesis that the improved osteoclastogenesis observed in the presence of the cytokine in *Fanca*^*−/−*^ OCs is due to increased NF-kB activity (Fig. 5A and B).

Thus, our observations indicate that in vitro osteoclastogenesis is impaired in *Fanca*^*−/−*^ cells as a consequence of impairment in both the p53 and NF-kB signaling pathways associated with Fanca loss-of-function, which can be rescued by TNFα exposure.

### Coculture of *Fanca*^*−/−*^ OCs with OB-like cells, mimicking in vivo conditions, rescues osteoclastogenesis

**Fig. 6:**
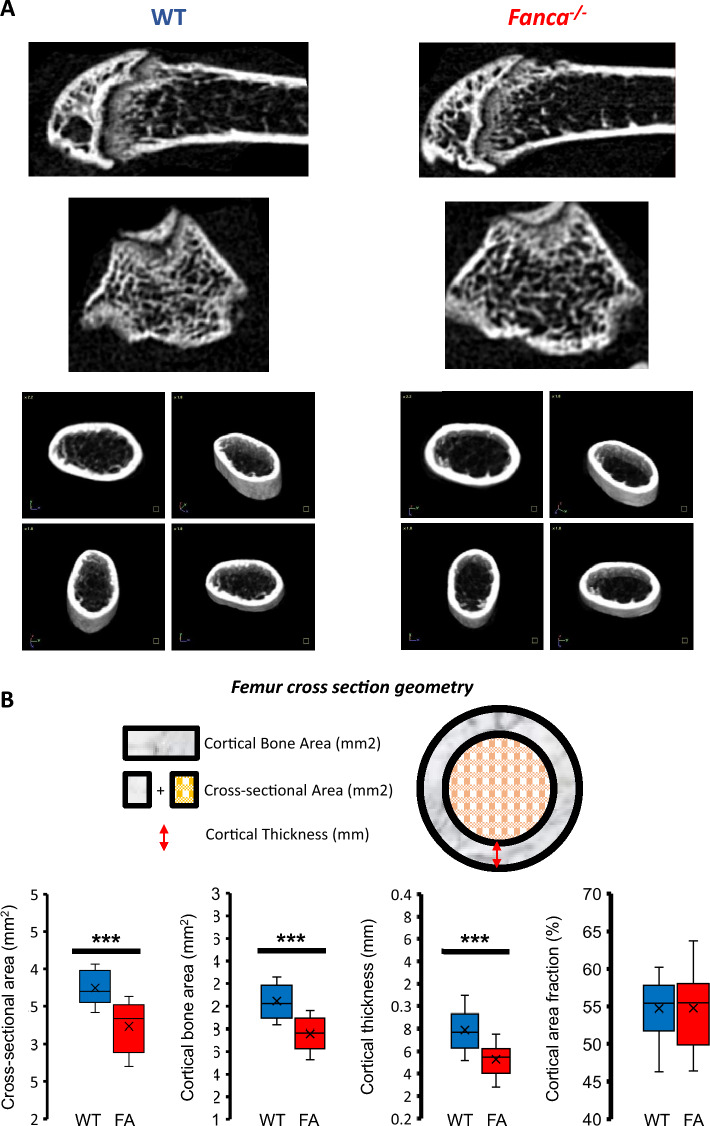
µCT analysis of femurs from Wt and Fanca^−/−^ mice. **A** Representative µCT images of the distal femur and femur section in WT and *Fanca*^*−/−*^ mice. **B** Comparison of femur cross-section geometry between WT and *Fanca*^*−/−*^ mice

Our previous in vitro data unveiling a defect in OC differentiation are in contrast with previously published observations showing, in FA patients and mouse models, an osteoporotic phenotype possibly associated with a defect in OB activity (Ref). Our *Fanca*^*−/−*^ mice developed normally, with no signs of skeletal abnormalities. However, a quantitative computed tomography analysis of the femur (Fig. 6A and Additional file 1: Fig. S4A) revealed that although the trabecular bone density was similar between the WT and *Fanca*^*−/−*^ mice (Additional file 1: Fig. S4B), the cortical bone in the *Fanca*^*−/−*^ mice was thinner, and its area was reduced (Fig. 6B). Thus, even in the absence of frank osteoporosis, the tomographic analysis is consistent with data from the literature that support a deficit in bone formation/accumulation and in contrast with the expectations of our in vitro data.

**Fig. 7:**
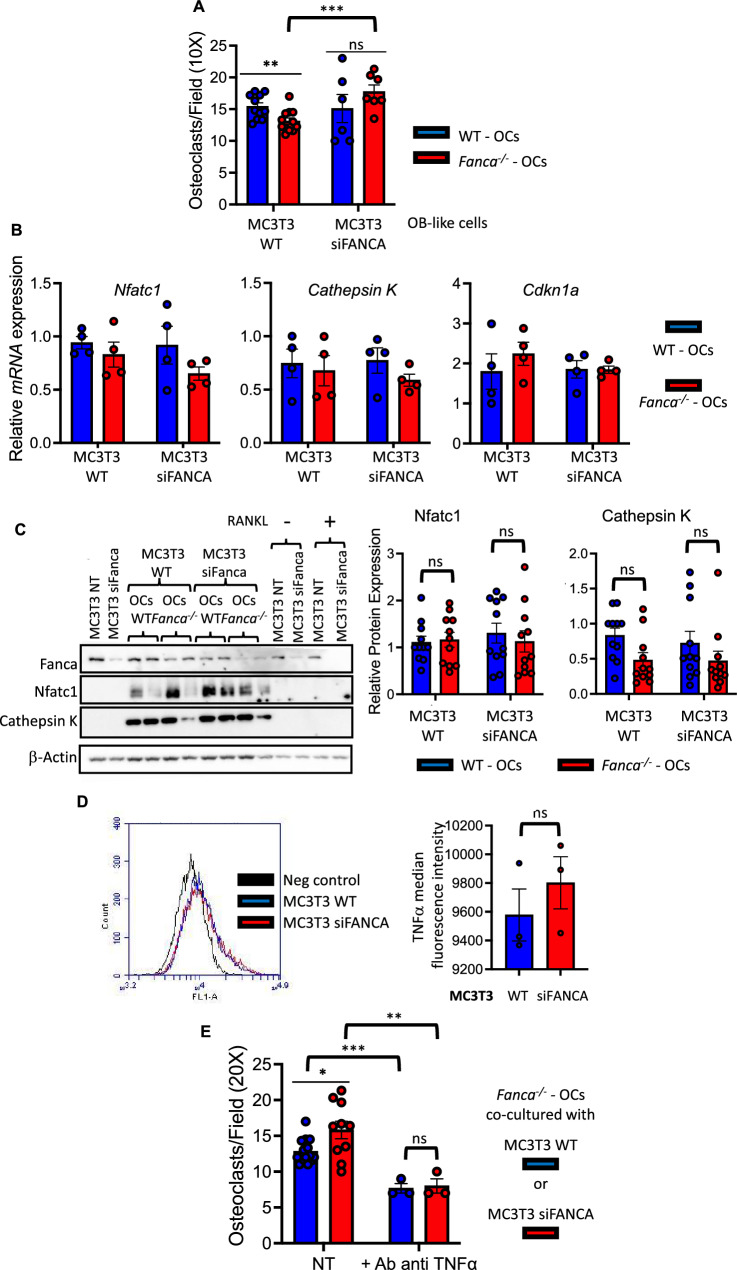
Coculture with the preosteoblastic cell line MC3T3 rescues impaired osteoclastogenesis induced by *Fanca* KO. **A** Number of OCs per field from WT and *Fanca*^*−/−*^ cells plated on WT or Fanca-depleted MC3T3 cells at day 4 of differentiation. **B** Relative *Nfatc1, Cathepsin K* and *Cdkn1a* expression evaluated by qRT-PCR in OCs from WT and *Fanca*^*−/−*^ mice cocultured with WT or Fanca-depleted MC3T3 cells at day 4 of differentiation. **C** Western blot showing Fanca, Nfatc1 and Cathepsin K in OCs from WT and *Fanca*^*−/−*^ mice at day 4 of differentiation plated on WT or Fanca-depleted MC3T3 cells. β-Actin was used as a loading control. Middle and right: Relative protein expression of Nfatc1 and Cathepsin K in OCs from WT and *Fanca*^*−/−*^ mice at day 4 of differentiation plated on WT or Fanca-depleted MC3T3 cells. **D** Relative TNFα level in the WT or siFanca-depleted MC3T3 cells as evaluated by FACS analysis. **E** Number of OCs per field from *Fanca*^*−/−*^ cells plated on WT or Fanca-depleted MC3T3 cells and left untreated (NT) or treated with an anti-TNFα neutralizing antibody (10 ng/ml) at day 4 of differentiation. Data are shown as the mean ± SEM. Each small circle represents an individual analyzed mouse or individual experiment (**D**). Statistical analysis was performed by t tests: *p < 0.05, **p < 0.01, ***p < 0.001, ****p < 0.0001

To shed light on the previous apparent parodox and given that TNFα exposure rescues *Fanca*^*−/−*^ osteoclastogenesis in vitro and that OC differentiation occurs on the bone endosteal surface under the control of OBs, which produce RANKL and TNFα, we decided to mimic this physiological setting in vitro. We layered WT or *Fanca*^*−/−*^ OC precursors on WT or siRNA Fanca-depleted MC3T3 murine cells, a widely accepted in vitro model of OBs [61]. The WT OC precursors cultured on the WT or siFanca-silenced MC3T3 cells differentiated into OCs (Fig. 7A). Compared to what we previously observed in cells cultured alone, the difference between *Fanca*^*−/−*^ and WT precursors was drastically reduced but still significant when *Fanca*^*−/−*^ cells were cultured on WT MC3T3 cells (Fig. 7A). Interestingly, when *Fanca*^*−/−*^ OC precursors were cocultured with Fanca-depleted OB-like MC3T3 cells, osteoclastogenesis was fully rescued (Fig. 7A). Importantly, as observed following TNFα exposure (Fig. 4), coculture conditions canceled the difference in the expression of *Nfatc1*, *Cathepsin K* and *Cdkn1a* between the WT and *Fanca*^*−/−*^ OCs at the mRNA and/or protein level (Fig. 7B and C). While an enzyme-linked immunosorbent assay (ELISA) of the culture medium did not reveal the presence of secreted TNFα (not shown), FACS analysis (Fig. 7D) demonstrated its presence at the cell membrane on both the WT and siFANCA-depleted MC3T3 cells. Notably, membrane-associated TNFα levels were slightly but systematically higher in Fanca-depleted MC3T3 cells than in Fanca-proficient MC3T3 cells. Next, to validate the role of TNFα in rescuing the defect in osteoclastogenesis in *Fanca*^*−/−*^ cells, we added a neutralizing TNFα antibody to WT or Fanca-depleted MC3T3 cells before adding WT or *Fanca*^*−/−*^ OC progenitors. OC progenitors from the same WT or *Fanca*^*−/−*^ mouse were cultured ‘alone’ in the absence or presence of TNFα, or seeded on WT or Fanca-depleted MC3T3 cells in the presence or absence of a neutralizing TNFα antibody. Exposure to TNFα (Additional file 1: Fig. S5A) or coculture with the WT or Fanca-depleted MC3T3 cells (Additional file 1: Fig. S5B) fostered and normalized *Fanca*^*−/−*^ osteoclastogenesis. Supporting the role of membrane-associated TNFα in rescuing *Fanca*^*−/−*^ osteoclastogenesis, the coculture of *Fanca*^*−/−*^ cells on Fanca-proficient or Fanca-deficient MC3T3 cells in the presence of neutralizing anti-TNFα significantly reduced their differentiation (Fig. 7E). Under similar culture conditions, the behavior of WT OCs remained the same (Additional file 1: Fig. S5C).

Thus, our analysis revealed that the observed defect in OC differentiation in *Fanca*^*−/−*^ cells can be rescued by TNFα and strongly supports the hypothesis that osteoclastogenesis in Fanca-deficient cells requires cytokines.

Thus, according to previously published observations, bone formation in FA appears impaired even if in vivo OC differentiation, which is clearly deficient per se, is rescued due to the cytokines present on the cells of the BM niche.

## Discussion

In this study, we demonstrated that the loss of Fanca function is associated with a defect in OC differentiation. Our data link the impairment of osteoclastogenesis to FA-associated dysregulation of p53–p21 and the NF-kB axis, both fully rescued in vitro by exposure to TNFα or by coculture with OB-like cells, which expose TNFα at their cytosolic membrane. Therefore, in vivo, the differentiation defect remains cryptic and hidden by the presence of OBs in the endosteum and/or TNFα in the local microenvironment. Bone histological analysis showed that the *Fanca*^*−/−*^ mice presented bone abnormalities in their cortical bone component compatible with osteoporotic pathology, as previously described.

Historically, all efforts made to elucidate the pathogenesis of FA have focused on the hematopoietic system at the HSC level, with relatively little attention given to the intrinsic differentiation process of the different lineages. Indeed, it has been proposed that BMF in FA relies on HSC attrition resulting from a combination of alterations in cell proliferation associated with unscheduled p53 [45, 47], MiTF [48] and/or MYC [47] expression and increased accumulation of DNA damage and genetic instability that arises when HSCs exit quiescence [62, 63], which pushes cells through differentiation, limiting self-renewal of potentially mutated HSCs [64, 65]. FA cells show altered expression, signaling and/or responses to several growth factors and proinflammatory cytokines, including TGFβ [44] and TNFα [43], whose origins and consequences are still poorly understood.

Moreover, evidence suggests that FANC pathway loss of function affects bone homeostasis and the skeletal system: FA patients display congenital abnormalities of the skeleton, mesenchymal tissue-derived malformations, and osteoporosis [49] partially recapitulated in embryos and/or adult *Fancc*^*−/−*^, *Fancg*^*−/−*^, *Fancd2*^*−/−*^ and *Fancc*^*−/−*^–*Fancg*^*−/−*^ double-KO (DKO) mice. Defects were related to altered osteogenic differentiation potential of bone marrow‐derived mesenchymal stem cells (BM-MSCs) but not defects in OC differentiation [49, 66–68].

Our analysis indicates that in our mouse model [69], the loss of *Fanca*^*−/−*^ affects hematopoiesis not only ‘quantitatively’, reducing the HSC pool but also functionally, altering the differentiation of OCs, which are a specific hematopoietic lineage. A ‘differentiation’ specific activity of Fanca has been reported for the process of megakaryopoiesis in *Fanca*^*−/−*^ mice [70] and for erythroid differentiation in human FANCA-deficient cells [62].

Our data validate and extend our knowledge of the negative consequences of the unscheduled activation of the p53–p21 axis in FA, which participates in HSC dysfunction [45] and defects in erythropoiesis [62]. However, p53 inhibition is not sufficient to completely correct deficient osteoclastogenesis in *Fanca*^*−/−*^ cells, as observed in response to TNFα. Exposure to the cytokine rescues the expression of p21 (as does p53 inhibition) as well as the expression of all targets of Nfatc1, the key driver of osteoclastogenesis. Thus, we propose that TNFα, by normalizing NF-kB signaling, promotes increased Nfatc1 activity on its canonical targets and ‘re-equilibrates’ p21 expression, allowing *Fanca*^*−/−*^ OC differentiation to proceed.

## Conclusions

We speculate that the overexpression of TNFα might represent a kind of adaptive response of the organism to ensure optimal OC differentiation despite the possible negative consequences of this cytokine on the behavior of other cell lines in FA, including on OB-mediated bone formation. A better understanding of the niche physiology in FA may help to establish supportive care for patients, delaying BMF, MDS and AML and supporting transplantation.

### Limitations of the study

Our study was conducted exclusively in mice and on one out of 23 FANC genes. Further studies are needed to evaluate whether depletion of any other protein of the FANC pathway causes similar abnormalities in OC physiology and in patients. Furthermore, additional studies are also required to determine if and how the inactivation of the FANC pathway affects the functionality of the OCs, i.e. their intrinsic ability to erode the bone matrix.

## Methods

### Mice and genotyping

*Fanca*^*−/−*^ mice on an FVB background were described previously [69]. The mice were a gift from F. Arwert (Free University Medical Center, Amsterdam, Netherlands) and have been routinely maintained in our laboratory for more than 10 years. *Fanca*^+*/–*^ mice were backcrossed with WT FVB/N mice (> 10 generations). As *Fanca*^*–/–*^ mice show severely reduced fertility, the WT and *Fanca*^*−/−*^ mice used for analysis correspond to siblings derived from crossbreeding of heterozygous mice. Mouse genotyping was performed using Dream-Taq Green DNA Polymerase (Thermo Fisher Scientific, #EP0711). The following primers (Eurogentec) were used: FANCA (forward 5′-GGATCAGGCCTCGAGGCTGG-3′, reverse 5′-TGCAGTAGCTCCTGTAGGCT-3′) and NEO (forward 5′-GACTGGGCACAACAGACAATCGGCT-3′, reverse 5′-TGATATTCGGCAAGCAGGCATCGCC-3′).

### Generation of OC cell culture

BM was harvested from mice by flushing tibias and femurs. Red blood cells were eliminated using EasySepTM Red Blood Cell Lysis Buffer (Stem Cell Technologies, #20110). BM cells were then cultured in αMEM with 10% FBS, 100 U/ml penicillin/50 μg/ml streptomycin and 25 ng/ml recombinant murine M-CSF (Peprotech, #315-02). After 48 h, viable progenitors were counted with Trypan Blue and seeded at a concentration of 75,000 cells/cm^2^ in αMEM with 10% FBS, 100 U/ml penicillin/50 μg/ml streptomycin and 50 ng/ml M-CSF and recombinant murine sRANK ligand (RANKL) (*Escherichia coli* derived, Peprotech, #315-11). The first OCs started to appear at day 3, and the medium was changed at day 4.

### TRAP staining

Mature OC cultures were fixed and stained for TRAP using a commercial kit [Acid Phosphatase, Leukocyte (TRAP) Kit, Sigma, #387A]. Briefly, cells were fixed using a fixative solution composed of 2.5 ml citrate solution, 6.5 ml acetone and 0.8 ml 37% formaldehyde for 30 s at room temperature (RT). Fifty microliters of Fast Garnet GBC Base Solution and fifty microliters of sodium nitrite were mixed for 30 s, left to stand at RT for 2 min and added to 4.5 ml of prewarmed deionized water (37 °C). Then, 50 µl of naphthol AS-BI phosphate solution, 200 µl of acetate solution and 200 µl of tartrate solution were added. The samples were incubated with the complete solution for 20 min at 37 °C. OCs were identified as TRAP-positive cells with 3 or more nuclei and a defined membrane.

### Immunofluorescence

Mature OCs were fixed in 4% formaldehyde for 15 min at RT and permeabilized in 0.1% Triton X-100 for 5 min at RT. Then, the cells were incubated with 1× phalloidin-iFluor 488 reagent (1:1000) (Abcam, #ab176753) for 90 min at 37 °C in the dark. For the 53BP1 analysis, the cells were fixed and permeabilized as previously mentioned and then incubated with a primary antibody anti-53BP1 (Abcam, #ab21083) at the same time as phalloidin. After 3 washes with PBS, the cells were incubated with an Alexa Fluor^®^594 donkey anti-rabbit secondary antibody for 30 min at 37 °C in the dark. Finally, the cells were incubated with a DAPI solution (1:500) for 3 min at RT and observed under a fluorescence microscope.

### Western blot

The collected cells were disrupted in lysis buffer (50 mM Tris-HCl pH 7.9, 40 mM NaCl, 1 mM MgCl_2_, 0.1% SDS, and 1% benzonase [Merck, 70746-3] with protease and phosphatase inhibitors [Roche, 05892791001 and 04906837001]). After incubation for 15 min at RT, protein concentrations were determined using the Bradford assay (Bio-Rad, 500-0006), and the samples were combined with 4× Laemmli buffer containing β-mercaptoethanol and denatured by boiling at 98 °C. The proteins (50 μg) were separated using SDS-PAGE, transferred onto a nitrocellulose membrane (GE Healthcare Life Sciences, Amersham Protran 0.2 μM NC, 10600001), and then exposed to the appropriate antibodies. The proteins were visualized using an enhanced chemiluminescence system (WesternBright ECL by Advansta, K-12045-050) and an ImageQuant LAS 4000 system (GE Healthcare Life Sciences). The following antibodies were used for Western blot analysis: rabbit anti-M-CSFR (Cell Signaling, #3152), mouse anti-RANK (Santa Cruz, #sc-374360), mouse anti-NFATc1 (BioLegend, #649607), mouse anti-Cathepsin K (Santa Cruz, #sc-48353), goat anti-p53 (R&D, #AF1355), rabbit anti-FANCA/FAA (Abcam, #ab272392), rabbit anti-phospho-NF-kB p65 (Ser536) (Cell Signaling, #3033), rabbit anti-RelB (Cell Signaling, #10544), rabbit anti-NF-kB1 p105/p50 (Cell Signaling, #13586), rabbit anti-NF-kB2 p100/p52 (Cell Signaling, #4882), mouse anti-MiTF (Abcam, #ab12039) and goat anti-β-Actin (Abcam, #ab8227). Red Ponceau staining was used as a loading control.

### qRT-PCR

Total RNA was isolated using the Maxwell^®^ RSC simplyRNA Cells Kit (Promega, #AS1390) and reverse-transcribed using the RevertAid First Strand cDNA Synthesis Kit (Thermo Scientific, #K1621). qPCR was performed using iTaq™ Universal SYBR^®^ Green Supermix (Bio-Rad, #1725120) and a CFX96 Touch Real-Time PCR Detection System (Bio-Rad). The following primers (Eurogentec) were used:

Rank: forward 5ʹ-GGACAACGGAATCAGATGTGGTC-3ʹ, Tm: 52.0 °C; reverse 5ʹ-CCACAGAGATGAAGAGGAGCAG-3ʹ, Tm: 51.6 °C;

M-Csfr: forward 5ʹ-TGGATGCCTGTGAATGGCTCTG-3ʹ, Tm: 51.6 °C; reverse 5ʹ-GTGGGTGTCATTCCAAACCTGC-3ʹ, Tm: 51.6 °C;

Nfatc1: forward 5ʹ-GGTGCCTTTTGCGAGCAGTATC-3ʹ, Tm: 52.1 °C; reverse 5ʹ-CGTATGGACCAGAATGTGACGG-3ʹ, Tm: 52.3 °C;

Oscar: forward 5ʹ-CGTGCTGACTTCACACCAACAG-3ʹ, Tm: 51.6 °C; reverse 5ʹ-AGTGCAAACCGCCAGGCAGATT-3ʹ, Tm: 51.6 °C;

Dc-Stamp: forward 5ʹ-TTTGCCGCTGTGGACTATCTGC-3ʹ, Tm: 51.7 °C; reverse 5ʹ-GCAGAATCATGGACGACTCCTTG-3ʹ, Tm: 51.9 °C;

Atp6v0d2: forward 5ʹ-ACGGTGATGTCACAGCAGACGT-3ʹ, Tm: 51.6 °C; reverse 5ʹ-CTCTGGATAGAGCCTGCCGCA-3ʹ, Tm: 53.1 °C;

Cathepsin K: forward 5ʹ-AGCAGAACGGAGGCATTGACTC-3ʹ, Tm: 51.7 °C; reverse 5ʹ-CCCTCTGCATTTAGCTGCCTTTG-3ʹ, Tm: 51.7 °C;

Trap: forward 5ʹ-GCGACCATTGTTAGCCACATACG-3ʹ, Tm: 51.6 °C; reverse 5ʹ-CGTTGATGTCGCACAGAGGGAT-3ʹ, Tm: 52.0 °C; 

p21 (Cdkn1a): forward 5ʹ-TCGCTGTCTTGCACTCTGGTGT-3ʹ, Tm: 52.0 °C; reverse 5ʹ-CCAATCTGCGCTTGGAGTGATAG-3ʹ, Tm: 51.9 °C.

### Transcriptomic analysis

Total RNA was extracted from the WT and *Fanca*^*−/−*^ progenitors and OCs as described in the “qRT-PCR” section. Global transcriptomic analysis was performed on total RNA using “Affymetrix Wtplus ClariomS Mouse”. Analysis was performed at the Plateforme de Génomique of the Cochin Institute (Inserm 1016-CNRS 8104), Paris Descartes University, Paris, FR.

### Chemicals, peptides, and recombinant proteins

We used recombinant murine TNFα (Peprotech, #315-01A), recombinant human TGF-β1 (HEK293 derived, Peprotech, #100-21), Nutlin-3 (Sigma, #6287), pifithtrin-α (Sigma, #P4359), butyrolactone I (Sigma, #203988) and human/mouse anti-TNF-alpha antibody (R&D Systems, #AF-410-NA) at the indicated concentrations. Antisense oligonucleotides directed against p21 were purchased from Eurogentec (5ʹ-ACA-TCA-CCA-GGA-TTG-GAC-ATG-GGG-GGG-GGG-3ʹ).

### Cell lines, culture conditions and siRNA transfection

The murine preosteoblastic cell line MC3T3-E1 Subclone 4 was purchased from ATCC (CRL-2593) and was routinely maintained in αMEM with 10% FBS, 100 U/ml penicillin/50 μg/ml streptomycin, 2 mM l-glutamine and 1 mM sodium pyruvate at 37 °C in a humidified atmosphere containing 5% CO_2_. For induction of OB differentiation under coculture conditions, the cell line was maintained in the same medium with 50 µg/ml l-ascorbic acid (Sigma #92902) and 4 mM β-glycerophosphate (Merck #G9422).siRNA directed against Fanca was purchased from Dharmacon (#L-058407-01-0005), and cells were transfected using Lipofectamine™ RNAiMAX transfection reagent (Thermo Fisher Scientific, #13779100) following the manufacturer’s instructions.

αMEM was added at the same time as OC precursors 24 h after siRNA transfection.

### FACS analysis

TNFα was evaluated by FACS analysis of MC3T3 cells 3 days after siRNA transfection. The cells were fixed for 15 min in 1% PFA/PBS, washed 1× in PBS, incubated for 30 min in the dark at RT at the appropriate dilution (1:100) with a FITC-conjugated anti-mouse TNFα antibody (BioLegend, #MP6-XT22) and washed 1× in PBS. The cells were then analyzed with a BD Accuri™ C6 Cytometer.

### Bone microarchitecture and histomorphometry

Cortical bone and trabecular bone microarchitecture assessment was performed on femurs using a high-resolution X-ray microcomputed tomography (micro-CT) system (Quantum FX Caliper, Life Sciences, Perkin Elmer). Micro-CT datasets were analyzed using the built-in multiplanar reconstruction tool Osirix 5.8 (Pixmeo). The reader was blinded to the status of the mouse (WT or *Fanca*^*−/−*^). For the cortical bone analysis of the femur, the following parameters were used: cross-sectional area (TtAr), cortical bone area (CtAr), cortical area fraction (CtAr/TtAr) and cortical thickness (CtTh). For the trabecular bone analysis of the femur, the following parameters were used: bone volume/total volume (BV/TV) ratio, trabecular number (TbN), trabecular separation (TbSp) and trabecular thickness (TbTh). Analyses were performed following published guidelines [71, 72].

### Statistical analysis

The results are expressed as the mean value ± SEM. Data were analyzed using Prism (GraphPad software, version 9). Differences were analyzed with unpaired and/or paired two-tailed t tests. Significance was set at 0.05.

## Supplementary Information

**Additional file 1: Figure S1.** A. Number of OCs for field, number of nuclei for OCand surface of the OCsat days 3, 4 and 5 of differentiation in 1-year-old mice. B. Relative *Fanca* expression evaluated by qRT-PCR in progenitors and OCs at day 4 of differentiation from WT and *Fanca*^*−/−*^ mice. Data are shown as the mean ± SEM. C. Western blot showing Fanca in OCs from WT and *Fanca*^*−/−*^ mice at days 4 and 5 of differentiation. b-Actin was used as a loading control. **Figure S2.** A. From left to right: relative *Rank, M-Csfr, Nfatc1, Dc-Stamp and Cathepsin K* expression evaluated by qRT-PCR in progenitors and OCs at day 4 of differentiation from 1-year-old WT and *Fanca*^*−/−*^ mice. Data are shown as the mean ± SEM. B. Western blot showing Nfatc1 in OCs from 1-year-old WT and *Fanca*^*−/−*^ mice at day 4 of differentiation. b-Actin was used as a loading control. Right: Relative protein expression of Nfatc1 in OCs from 1-year-old WT and *Fanca*^*−/−*^ mice at day 4 of differentiation. C. Western blotand quantificationshowing Mitf expression in OCs from WT and *Fanca*^*−/−*^ mice at day 4 of differentiation. **Figure S3.** A. Number of OCs for field, number of nuclei for Csand surface of the OCsat day 4 of differentiation in the WT and *Fanca*^*−/−*^ cells left untreated or treated with TGFb. **Figure S4.** A. Representative µCT images of the distal femur and femur sections in WT and *Fanca*^*−/−*^ mice. B. Comparison of trabecular status in bone from WT and *Fanca*^*−/−*^ mice. **Figure S5.** A. Number of OCs per field from WT and *Fanca*^*−/−*^ cells cultured in the absence or presence of TNFα on day 4 of differentiation. B. Number of OCs per field from the WT and *Fanca*^*−/−*^ cells plated on WT or Fanca-depleted MC3T3 cells and treated or not with an inhibitory anti- TNFα antibody at day 4 of differentiation. C. Number of OCs per field from the WT cells plated on WT or Fanca-depleted MC3T3 cells and treated or not with an inhibitory anti- TNFα antibody at day 4 of differentiation. **Figure S6.** Uncropped gel images used for representative Western blot.

## Data Availability

All data generated or analyzed during this study are included in this published article and its Additional information files. Indeed, data from each single mouse are presented as ‘dots’ in each figure. However, the datasets used and/or analyzed during the current study are available from the corresponding author on reasonable request.

## Declarations

### Study approval

This project was officially approved by the Animal Experimentation Ethics Committee (no. 26) of Gustave Roussy and conducted in accordance with French laws and regulations.
