# Supplementary material for: Fanca deficiency is associated with alterations in osteoclastogenesis that are rescued by TNFα
Source: Cell Biosci. 2023 Jun 24;13:115. doi: 10.1186/s13578-023-01067-7 (PMC10290407; doi:10.1186/s13578-023-01067-7)
Supplement: Supplementary file 1 — Additional file 1: Figure S1. A. Number of OCs for field, number of nuclei for OCand surface of the OCsat days 3, 4 and 5 of differentiation in 1-year-old mice. B. Relative Fanca expression evaluated by qRT-PCR in progenitors and OCs at day 4 of differentiation from WT and Fanca−/− mice. Data are shown as the mean ± SEM. C. Western blot showing Fanca in OCs from WT and Fanca−/− mice at days 4 and 5 of differentiation. b-Actin was used as a loading control. Figure S2. A. From left to right: relative Rank, M-Csfr, Nfatc1, Dc-Stamp and Cathepsin K expression evaluated by qRT-PCR in progenitors and OCs at day 4 of differentiation from 1-year-old WT and Fanca−/− mice. Data are shown as the mean ± SEM. B. Western blot showing Nfatc1 in OCs from 1-year-old WT and Fanca−/− mice at day 4 of differentiation. b-Actin was used as a loading control. Right: Relative protein expression of Nfatc1 in OCs from 1-year-old WT and Fanca−/− mice at day 4 of differentiation. C. Western blotand quantificationshowing Mitf expression in OCs from WT and Fanca−/− mice at day 4 of differentiation. Figure S3. A. Number of OCs for field, number of nuclei for Csand surface of the OCsat day 4 of differentiation in the WT and Fanca−/− cells left untreated or treated with TGFb. Figure S4. A. Representative µCT images of the distal femur and femur sections in WT and Fanca−/− mice. B. Comparison of trabecular status in bone from WT and Fanca−/− mice. Figure S5. A. Number of OCs per field from WT and Fanca−/− cells cultured in the absence or presence of TNFα on day 4 of differentiation. B. Number of OCs per field from the WT and Fanca−/− cells plated on WT or Fanca-depleted MC3T3 cells and treated or not with an inhibitory anti- TNFα antibody at day 4 of differentiation. C. Number of OCs per field from the WT cells plated on WT or Fanca-depleted MC3T3 cells and treated or not with an inhibitory anti- TNFα antibody at day 4 of differentiation. Figure S6. Uncropped gel images used for representative Weste [file 13578_2023_1067_MOESM1_ESM.pdf]

Fanca deficiency is associated with alterations in osteoclastogenesis that are rescued by TNFα

Alessia Oppezzo[1,2,3,£], Lovely Monney[1,2,3], Henri Kilian[4], Lofti Slimani[4], Frédérique Maczkowiak-Chartois[1,2,3] and Filippo Rosselli[1,2,3,*]

[1]CNRS UMR9019, Équipe labellisée La Ligue contre le Cancer, Villejuif, France.
[2]Gustave Roussy Cancer Center, Villejuif, France.
[3]Université Paris Saclay, Orsay, France.
[4]Université de Paris, URP2496 Pathologies, Imagerie et Biothérapies Orofaciales et Plateforme Imagerie du Vivant (PIV), FHU-DDS-net, Dental School, Montrouge, France.
£ Present address: IFOM ETS, the AIRC Institute of Molecular Oncology, Milan, Italy

Supplemental figures

Supplementary Figure 1.

A. Number of OCs for field (left), number of nuclei for OC (middle) and surface of the OCs (right) at days 3, 4 and 5 of differentiation in 1-year-old mice. **B.** Relative *Fanca* expression evaluated by qRT–PCR in progenitors and OCs at day 4 of differentiation from WT and *Fanca*$^{-/-}$ mice. Data are shown as the mean ± SEM. **C.** Western blots showing Fanca in OCs from WT and *Fanca*$^{-/-}$ mice at days 4 and 5 of differentiation. β-Actin was used as a loading control.

Supplementary Figure 2.

A. From left to right: relative *Rank, M-Csfr, Nfatc1, Dc-Stamp and Cathepsin K* expression evaluated by qRT–PCR in progenitors and OCs at day 4 of differentiation from 1-year-old WT and *Fanca*$^{-/-}$ mice. Data are shown as the mean ± SEM. **B.** Western blot showing Nfatc1 in OCs from 1-year-old WT and *Fanca*$^{-/-}$ mice at day 4 of differentiation. β-Actin was used as a loading control. Right: Relative protein expression of Nfatc1 in OCs from 1-year-old WT and *Fanca*$^{-/-}$ mice at day 4 of differentiation. **C.** Western blot (right) and quantification (left) showing Mitf expression in OCs from WT and *Fanca*$^{-/-}$ mice at day 4 of differentiation.

Supplementary Figure 3.

A. Number of OCs for field (left), number of nuclei for Cs (middle) and surface of the OCs (right) at day 4 of differentiation in the WT and *Fanca*$^{-/-}$ cells left untreated or treated with TGFβ (10 ng/ml).

Supplementary Figure 4.

A. Representative µCT images of the distal femur and femur sections in WT and *Fanca*$^{-/-}$ mice.

B. Comparison of trabecular status in bone from WT and *Fanca*$^{-/-}$ mice.

Supplementary Figure 5.

A. Number of OCs per field from WT and *Fanca*$^{-/-}$ cells cultured in the absence or presence of TNFα on day 4 of differentiation.

B. Number of OCs per field from the WT and *Fanca*$^{-/-}$ cells plated on WT or Fanca-depleted MC3T3 cells and treated or not with an inhibitory anti-TNFα antibody at day 4 of differentiation.

C. Number of OCs per field from the WT cells plated on WT or Fanca-depleted MC3T3 cells and treated or not with an inhibitory anti-TNFα antibody at day 4 of differentiation.

Supplemental Figure 6.

Uncropped gel images used for representative Western blots.

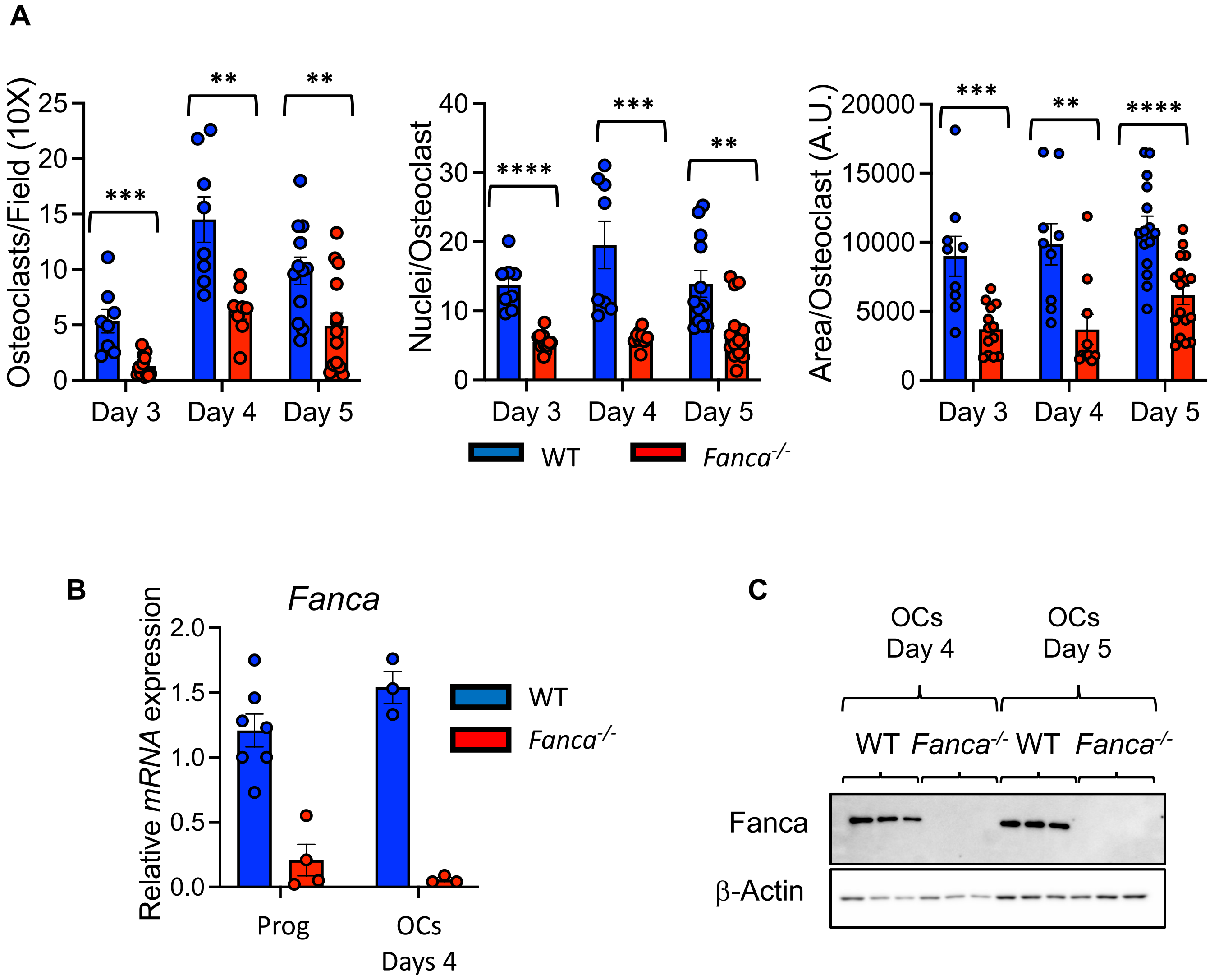

Supplementary Figure 1

A

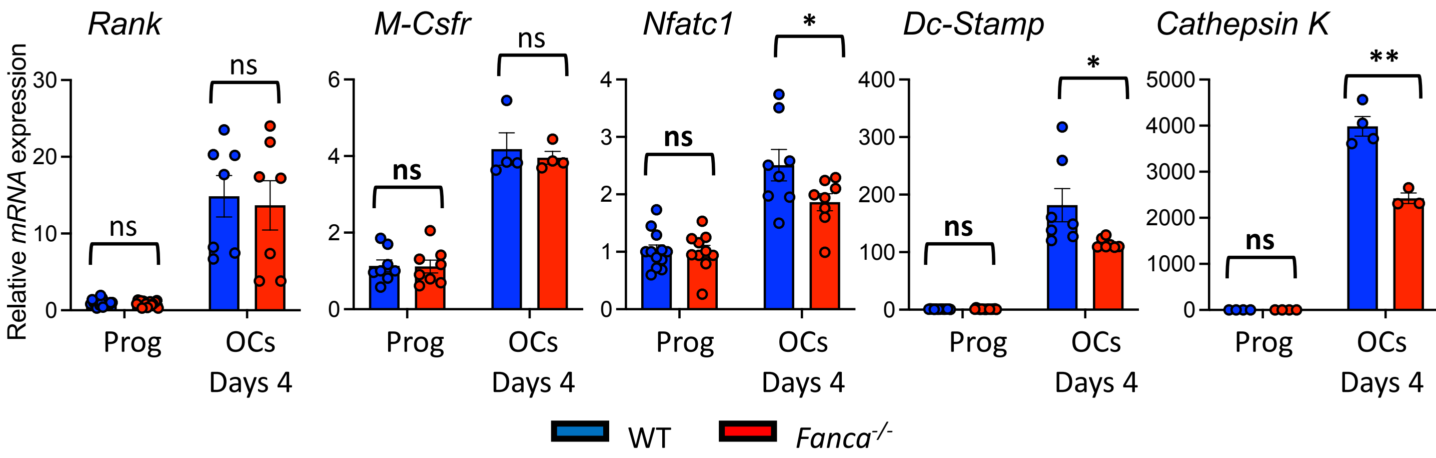

B

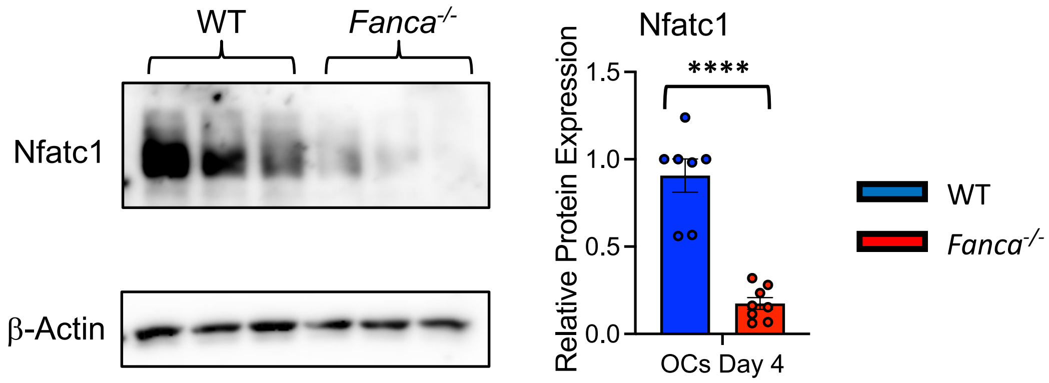

C

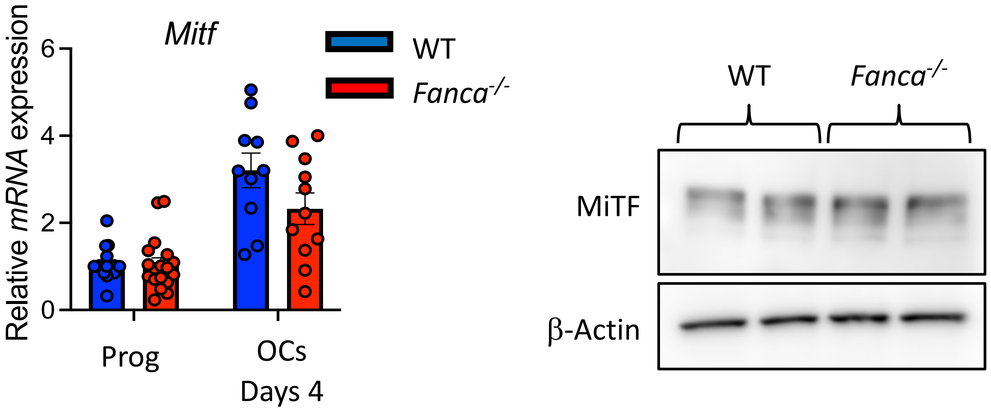

Supplementary Figure 2

A

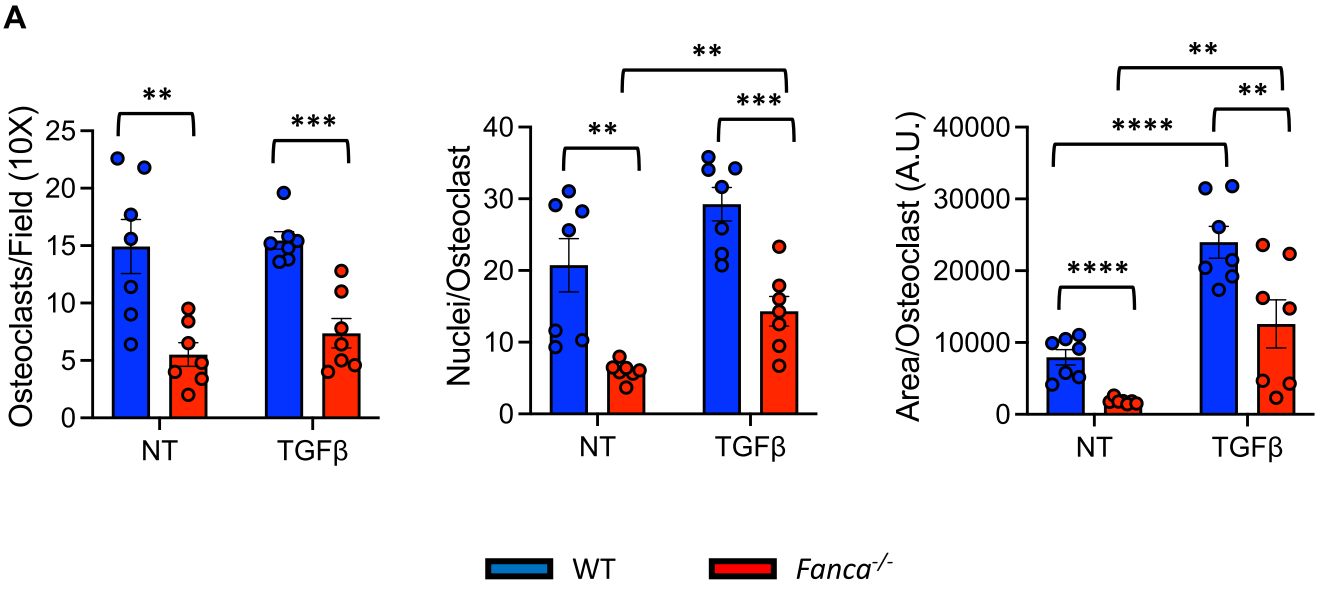

Supplementary figure 3

A

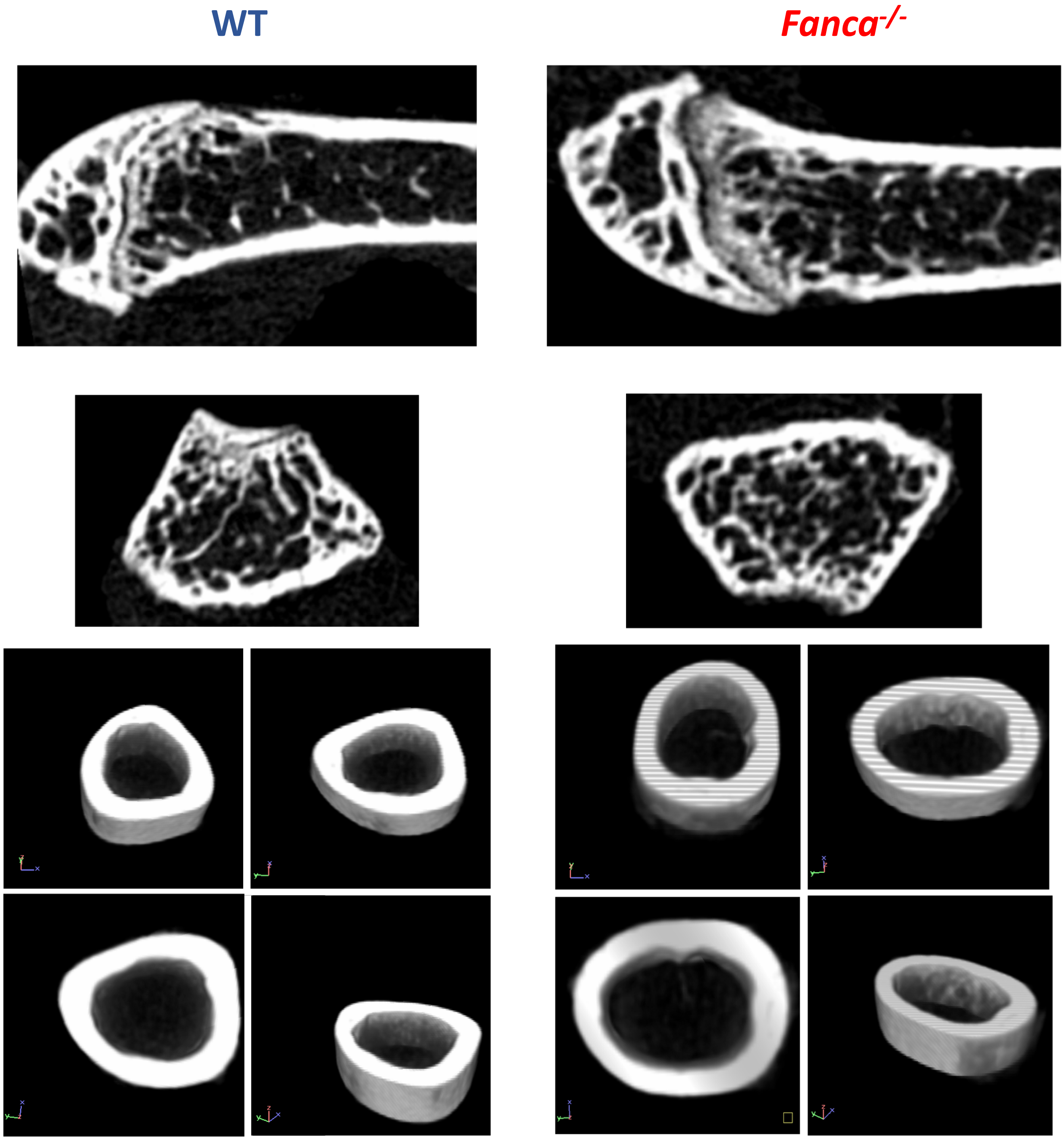

B

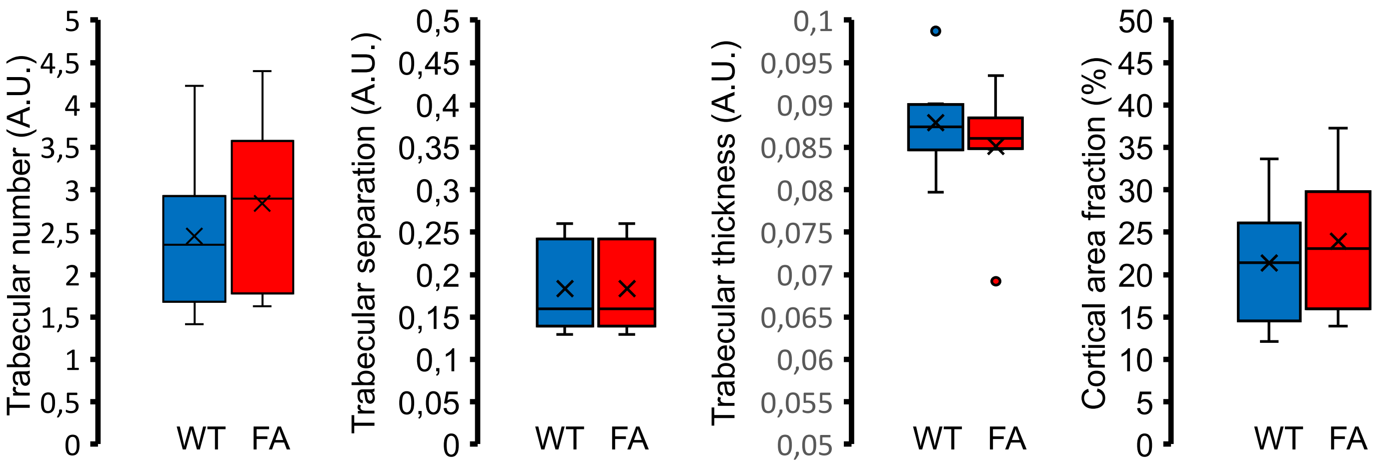

Supplementary figure 4

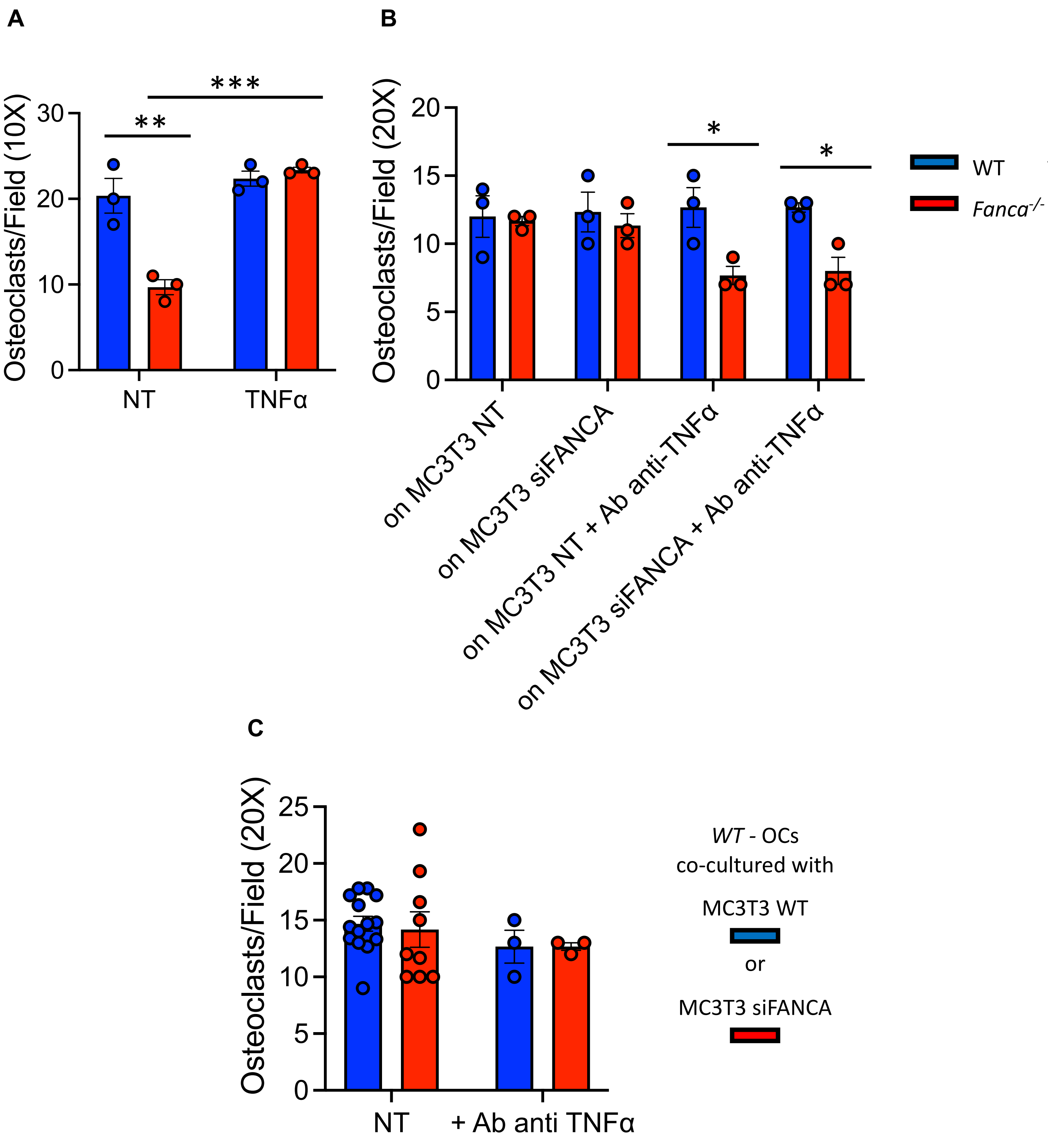

Supplementary figure 5

FIG.2D

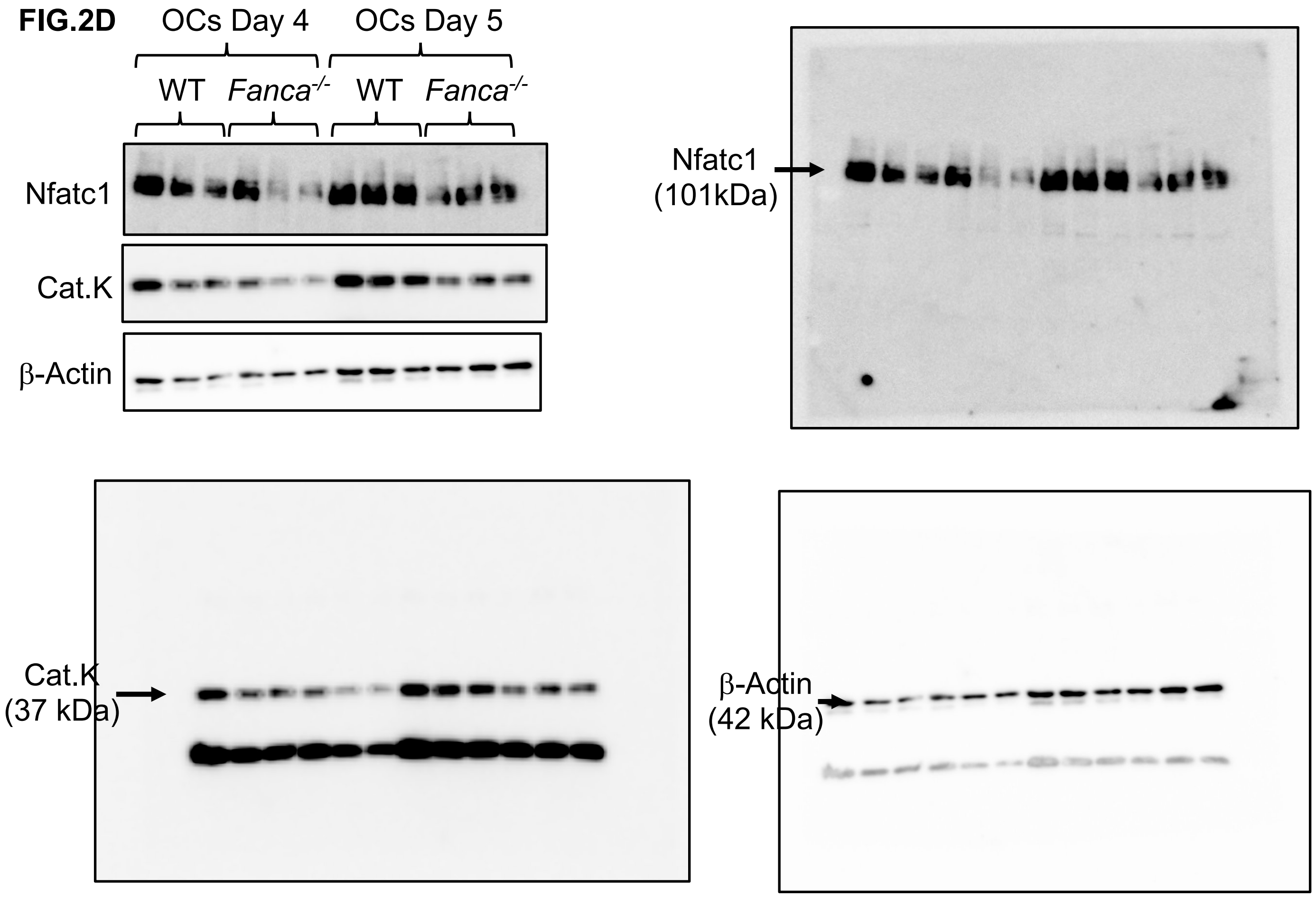

FIG.4C

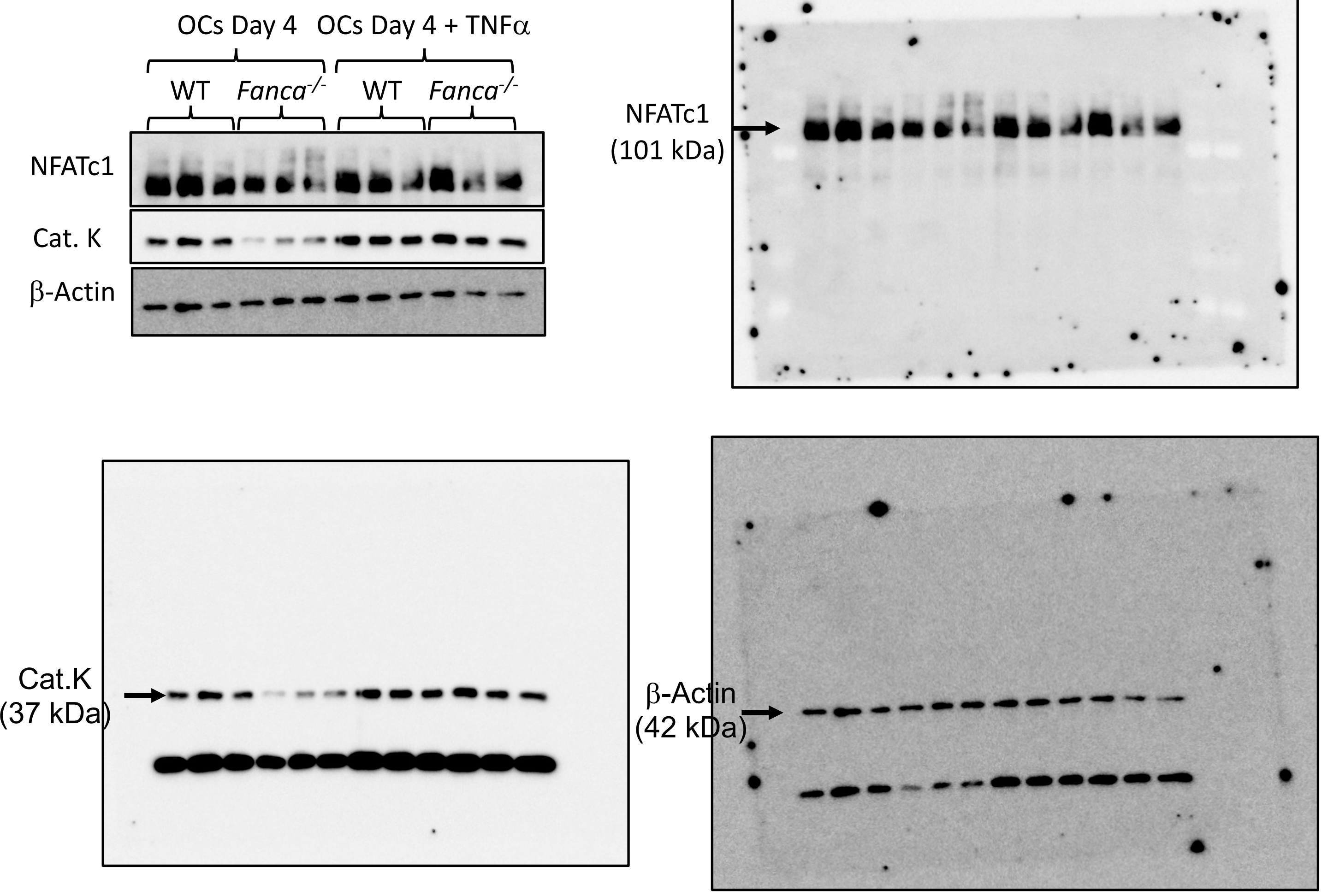

Supplementary figure 6

FIG.5A

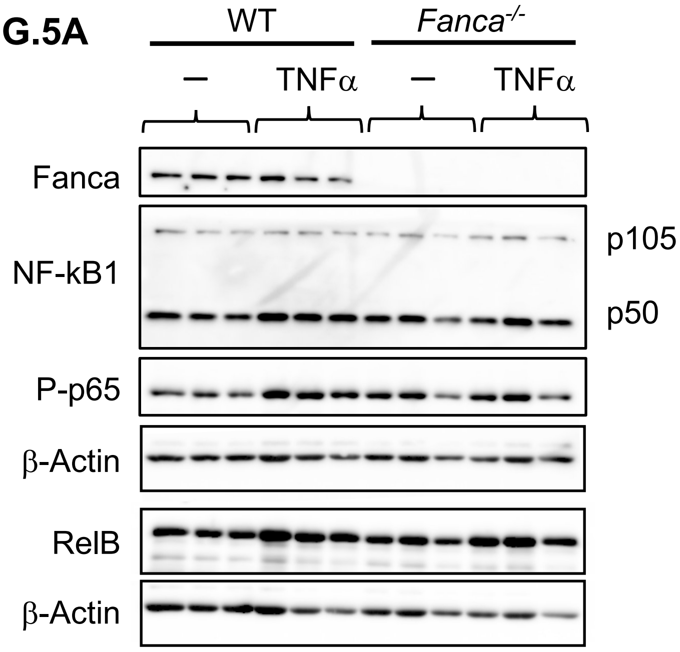

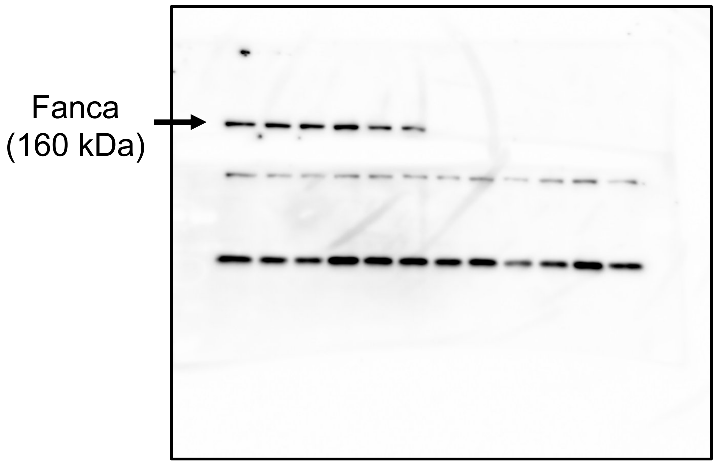

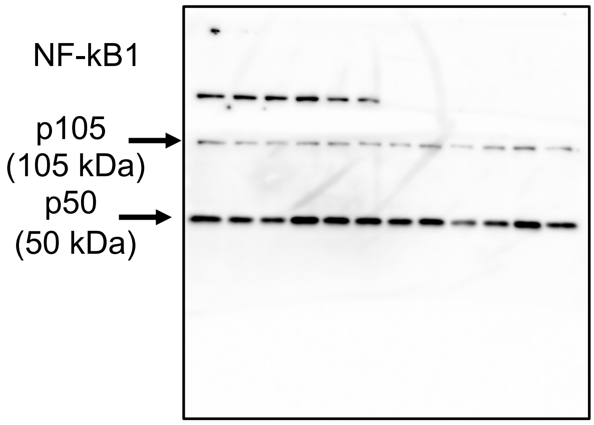

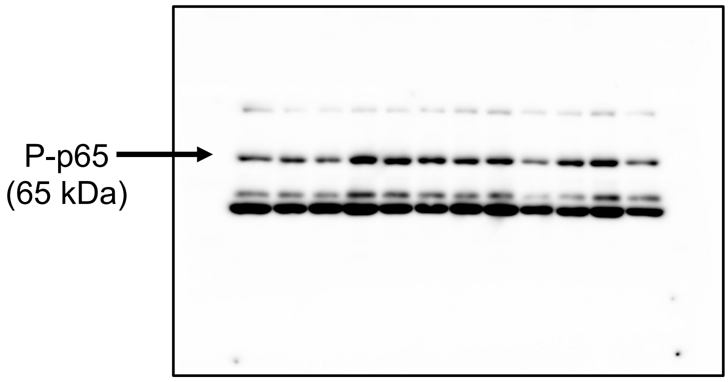

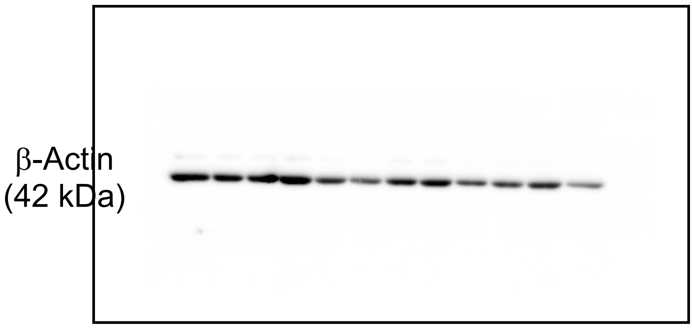

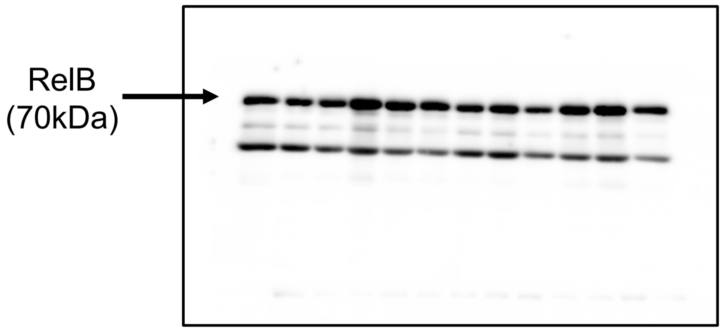

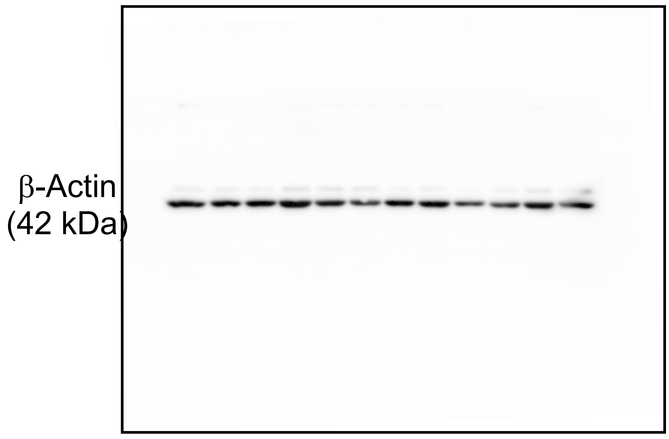

Supplementary figure 6

FIG7C

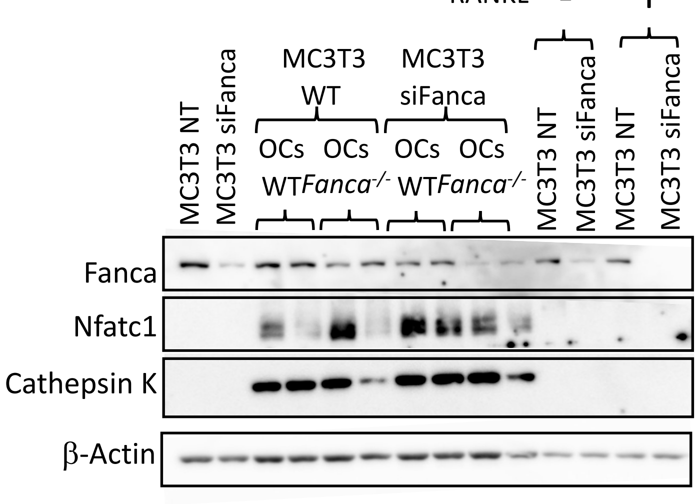

Fanca (160 kDa)

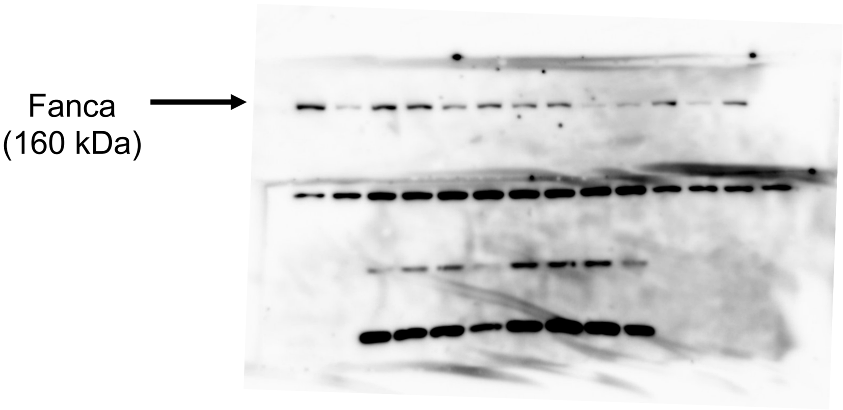

NFATc1 (101 kDa)

Cat.K (37 kDa)

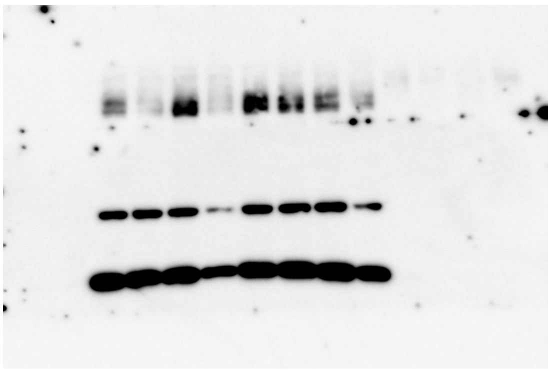

β-Actin (42 kDa)

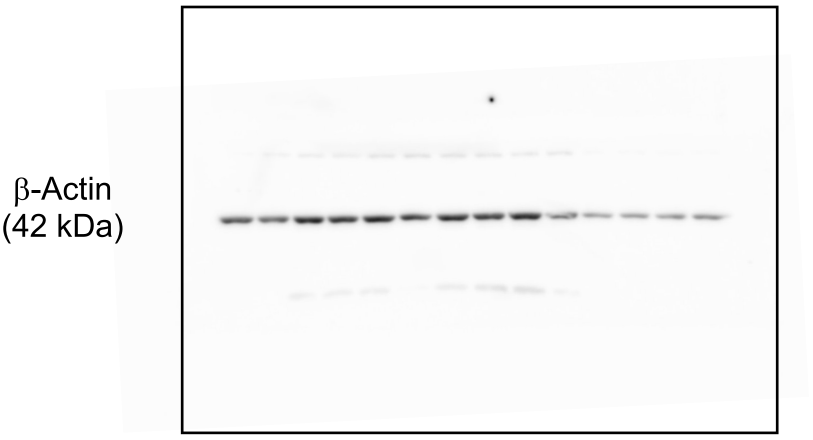

Supplementary figure 6

SUPPL1C

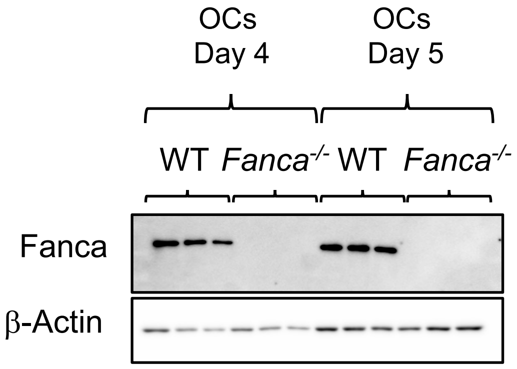

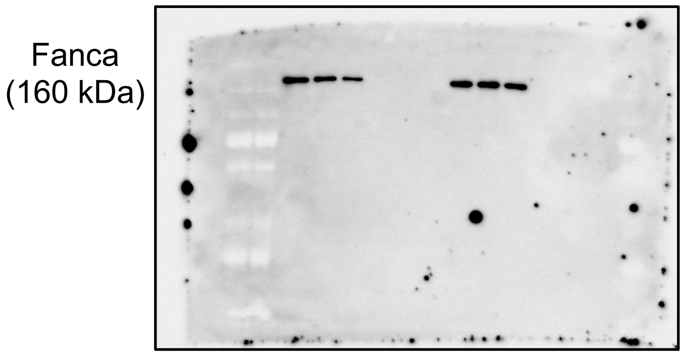

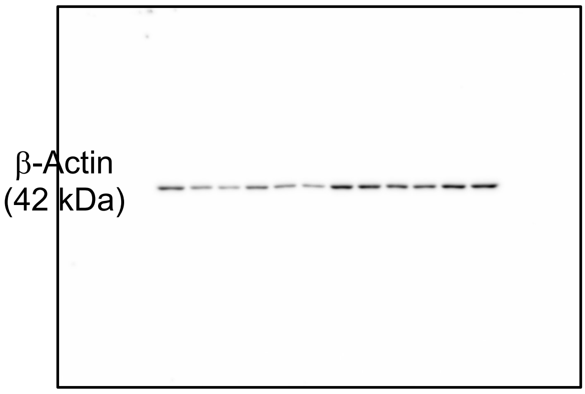

SUPPL2B

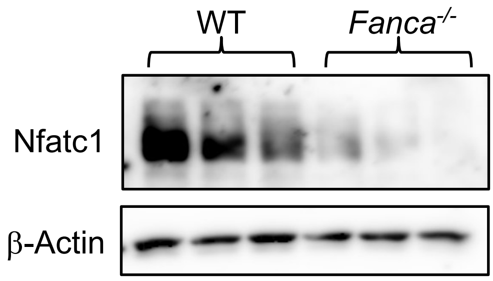

SUPPL2C

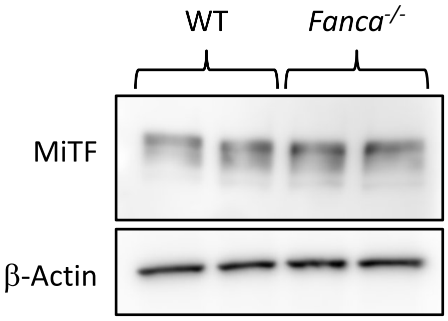

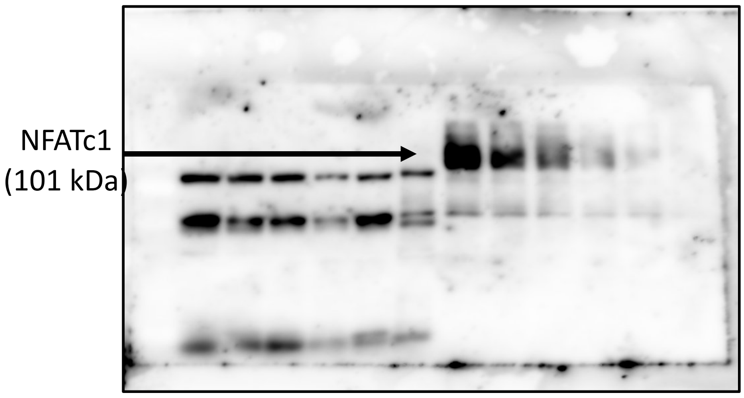

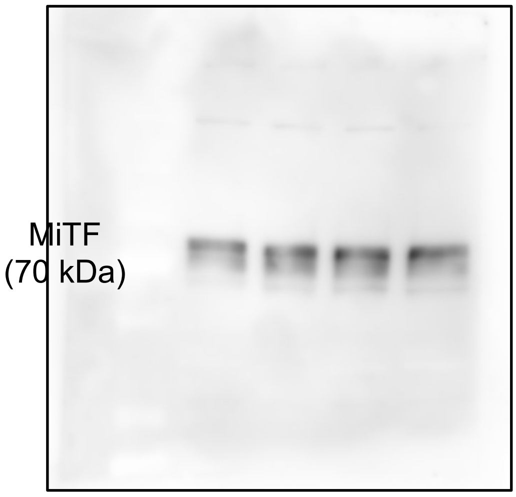

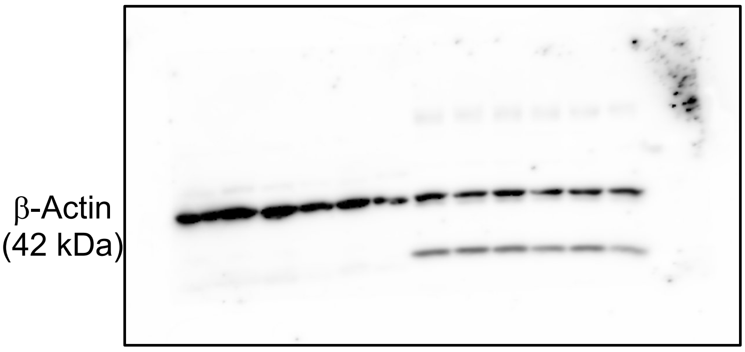

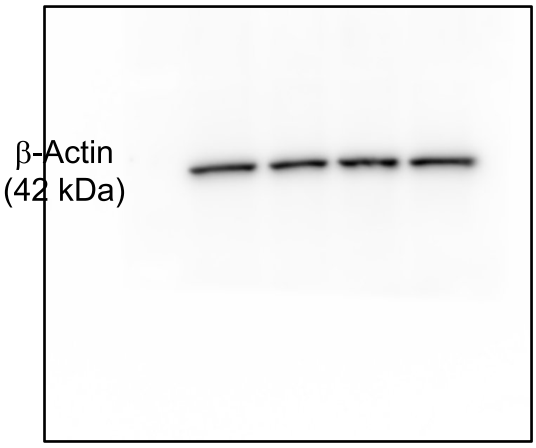

Supplementary figure 6
